# Highly Specialized Carbohydrate Metabolism Capability in *Bifidobacterium* Strains Associated with Intestinal Barrier Maturation in Early Preterm Infants

**DOI:** 10.1128/mbio.01299-22

**Published:** 2022-06-13

**Authors:** Bing Ma, Sripriya Sundararajan, Gita Nadimpalli, Michael France, Elias McComb, Lindsay Rutt, Jose M. Lemme-Dumit, Elise Janofsky, Lisa S. Roskes, Pawel Gajer, Li Fu, Hongqiu Yang, Mike Humphrys, Luke J. Tallon, Lisa Sadzewicz, Marcela F. Pasetti, Jacques Ravel, Rose M. Viscardi

**Affiliations:** a Institute for Genome Sciences, University of Maryland School of Medicine, Baltimore, Maryland, USA; b Department of Microbiology and Immunology, University of Maryland School of Medicine, Baltimore, Maryland, USA; c Department of Pediatrics, University of Maryland School of Medicine, Baltimore, Maryland, USA; d Department of Epidemiology, University of Maryland School of Medicine, Baltimore, Maryland, USA; e Center for Vaccine Development and Global Health, University of Maryland School of Medicine, Baltimore, Maryland, USA; Rutgers, The State University of New Jersey

**Keywords:** preterm infant, gut microbiome, leaky gut, intestinal barrier maturation, human milk oligosaccharides, *Bifidobacterium*

## Abstract

“Leaky gut,” or high intestinal barrier permeability, is common in preterm newborns. The role of the microbiota in this process remains largely uncharacterized. We employed both short- and long-read sequencing of the 16S rRNA gene and metagenomes to characterize the intestinal microbiome of a longitudinal cohort of 113 preterm infants born between 24^0/7^ and 32^6/7^ weeks of gestation. Enabled by enhanced taxonomic resolution, we found that a significantly increased abundance of Bifidobacterium breve and a diet rich in mother’s breastmilk were associated with intestinal barrier maturation during the first week of life. We combined these factors using genome-resolved metagenomics and identified a highly specialized genetic capability of the *Bifidobacterium* strains to assimilate human milk oligosaccharides and host-derived glycoproteins. Our study proposes mechanistic roles of breastmilk feeding and intestinal microbial colonization in postnatal intestinal barrier maturation; these observations are critical toward advancing therapeutics to prevent and treat hyperpermeable gut-associated conditions, including necrotizing enterocolitis (NEC).

## INTRODUCTION

Early preterm neonates are particularly vulnerable to life-threatening events and routinely require intensive care and medical intervention to survive (1). The physiological immaturity of their gastrointestinal (GI) tract is commonly associated with deficiencies in barrier functions that result in a clinical syndrome known as “leaky gut” (2–5). Under leaky gut conditions, the bacteria and bacterial products normally confined to the intestinal lumen are able to translocate into the peripheral circulation through the hyperpermeable epithelial barrier, which could lead to the widespread invasion of the intestinal epithelium and gut lamina propria, mucosal inflammation, epithelial cell damage, intestinal necrosis, systemic infection, and, ultimately, multiorgan failure and death (4, 6, 7). Necrotizing enterocolitis (NEC) is a prominent bacterial translocation-associated GI condition that affects 7 to 10% of preterm neonates or 1 to 5% of all neonatal intensive care unit (NICU) admissions, with a devastating mortality rate as high as 50% (8–12). The early detection of an aberrant leaky gut and early intervention to limit intestinal injury are of paramount importance to reduce the incidence of subsequent complications, including NEC (12, 13).

A functional intestinal barrier combines a physical barrier that encompasses chemical, immunological, and microbiological components (14). We and others have found that the first week of life (day 8 ± 2 after birth) is a critical window during which the most rapid postnatal intestinal maturation occurs (15–17). More importantly, these previous studies demonstrated that intestinal barrier function, which develops mostly *in utero* in term infants, can be improved postnatally. They also showed that intestinal barrier maturation does not occur at the same rate, with ~40% of preterm neonates (<33 weeks of gestation) failing to develop a functional intestinal barrier within the first 2 weeks of life (15, 16). Determining the factors that drive this developmental disparity will inform early detection and novel therapeutic strategies to promote intestinal barrier maturation.

Efforts to characterize the microbiological factors that are associated with intestinal barrier maturation have thus far yielded unsatisfactory results (18). There are no microbial biomarkers predictive of intestinal development. A major limitation is the use of partial 16S rRNA gene sequences to evaluate the taxonomic composition of the gut microbiota. Short sequences lack the phylogenetic signal necessary to describe the taxonomic composition at the species or even the genus level. Many of the PCR primers used to amplify variable regions of the 16S rRNA gene fail to amplify members of the genus *Bifidobacterium* (19–21). *Bifidobacterium* species are known to be frequent colonizers of the infant gut (22), are considered to play beneficial roles in intestinal development, and influence the maturation of the neonatal gut, potentially through stimulating colonic epithelial proliferation, modulation of host defense responses, and protection against bacterial infections (23, 24). Investigating *Bifidobacterium* and other bacterial groups predictive of early intestinal development and maturation is of pivotal importance.

In this study, we sought to characterize the role of the early assembly of the infant gut microbiota and its metabolism in postnatal intestinal barrier maturation. We build upon the results of previous studies (15, 16), using an expanded cohort (*n* = 113) of early preterm neonates (24 weeks and 0 day to 32 weeks and 6 days of gestation) from whom stool samples were collected daily up to 21 days after birth. High-resolution approaches were applied to characterize the composition of the developing gut microbiota with a substantially enhanced taxonomic resolution, including *Bifidobacterium* species, which we identified as the microbial biomarker associated with postnatal intestinal barrier maturation within the first week of life. Whole-community metagenomes using both short- and long-read sequences provided a detailed characterization of the genetic content of these *Bifidobacterium* species, which were shown to have distinct genetic features affording complete carbohydrate-foraging capabilities, including human milk oligosaccharides (HMOs) and host-derived glycoprotein. The presence of specific strains of *Bifidobacterium* may inform the early detection of aberrant intestinal permeability (IP). Supplementation with these bifidobacterial strains could be leveraged in novel intervention strategies for the prevention of leaky gut and its devastating sequelae in preterm newborns.

## RESULTS

### Clinical cohort.

**FIG 1:**
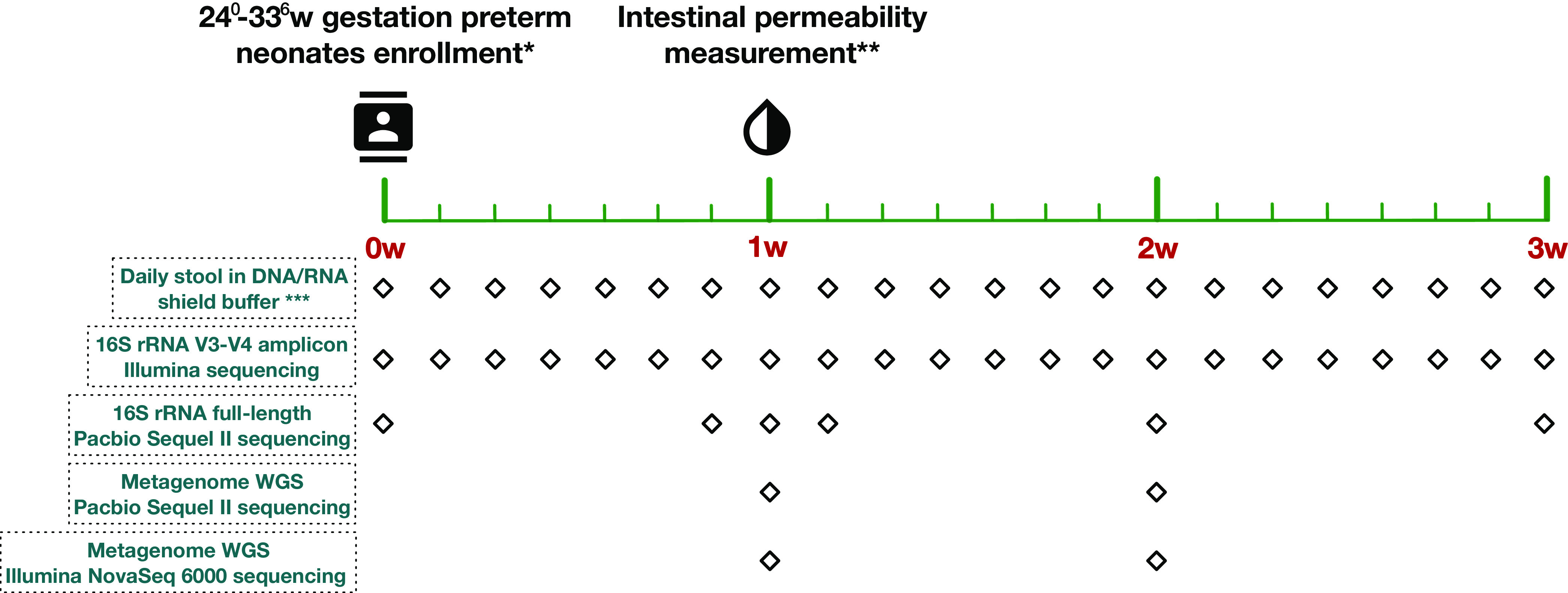
Study design. *, demographic, clinical, and nutritional information was collected for each enrolled preterm neonate. Inclusion criteria include 24^0^ to 32^6^ weeks and <4 days of age. Exclusion criteria include nonviable or planned withdrawal of care, severe asphyxia, chromosome abnormalities, cyanotic congenital heart disease, intestinal atresia or perforation, abdominal wall defects, significant GI dysfunction, and galactosemia or other forms of galactose intolerance. **, intestinal permeability was measured using the urine non-metabolized sugar probes lactulose and rhamnose at days 7 to 10 after birth. ***, stool specimens were collected daily at every stooling event, stored in storage buffer, and archived at −80°C. WGS, whole-genome sequencing.

We examined a prospective cohort of 113 preterm infants at 24^0/7^ to 32^6/7^ weeks of gestation, including 37 subjects described in a previous analysis (see Table S1 in the supplemental material). Fecal samples were collected daily until postnatal day 21 or discharge from the neonatal intensive care unit (NICU) (Fig. 1). The mean gestational age (GA) of infants at birth was 29.9 ± 2.3 weeks. A total of 28 infants (24.8%) were <28 weeks GA, and 85 (75.2%) were 28^0/7^ to 32^6/7^ weeks GA. The mean birth weight was 1,381 g (±415 g); 66 (58.4%) newborns were classified as having a very low birth weight (VLBW) (birth weight of <1,500 g), and 26 (23.0%) were classified as having an extremely low birth weight (ELBW) (<1,000 g).

10.1128/mbio.01299-22.7 TABLE S1 Clinical metadata of the 113 early preterm infant subjects in this study. Download Table S1, XLSX file, 0.02 MB. Copyright © 2022 Ma et al. 2022 Ma et al. https://creativecommons.org/licenses/by/4.0/ This content is distributed under the terms of the Creative Commons Attribution 4.0 International license.

Intestinal permeability (IP) was determined 7 to 10 days after birth, when rapid intestinal barrier maturation normally takes place (15, 16). IP was calculated as the ratio of two enterally administered sugar probes, lactulose (La) and rhamnose (Rh), markers of intestinal paracellular and transcellular pathways, respectively (25, 26). IP ranged between 0.001 and 0.394, with an average of 0.07 ± 0.007 (mean ± standard error [SE]), and was not significantly different among postnatal days 7 to 10 (Fig. S1A). High IP was defined by an La/Rh ratio of >0.05, as validated and applied previously (16). Of the 113 subjects, 48 (42.5%) were found to have high IP. Infants <28 weeks GA were more likely to have elevated IP (*n* = 18) than infants 28^0/7^ to 32^6/7^ weeks GA (64.3% versus 35.3% [*P* < 0.01]).

10.1128/mbio.01299-22.1 FIG S1 Intestinal permeability (IP) and cohort clinical information. (A) Notched box plot of IP for early preterm subjects (gestational age [GA], <33 weeks of gestation). Subjects were categorized by the IP measurement day between study days 7 and 10 and by IP category. The top and bottom of the box are the lower and upper quartiles, and the band near the middle of the box represents the median. The width of the notch can be used to roughly compare two distributions. For example, two distributions without overlapping notch regions can be roughly considered to be significantly different from each other (1). IP was measured by the nonmetabolized sugar probes lactulose and rhamnose. High IP was defined as an La/Rh ratio of >0.05, as validated and applied previously (2). (B) Correlation matrix visualization of the subjects’ physiological age. R package correlograms (corrgram) were used to visualize the correlation matrices. The Pearson correlation method was used to calculate correlations. Abbreviations: PMA at dosing, postmenstrual age calculated at the dosing day when IP was measured; PMA at enrollment, postmenstrual age at the enrollment day taking place within 1 to 4 days after birth; BW, birth weight; body weight at dosing, subject weight measured at the dosing day when IP was measured. Download FIG S1, PDF file, 0.1 MB. Copyright © 2022 Ma et al. 2022 Ma et al. https://creativecommons.org/licenses/by/4.0/ This content is distributed under the terms of the Creative Commons Attribution 4.0 International license.

### Postmenstrual age and mother’s own breastmilk feeding are associated with intestinal permeability in early preterm neonates.

Among the collected demographic and maternal variables for each infant, four host factors were observed to be inversely related to IP, including GA, postmenstrual age (PMA) corresponding to chronological age and GA, birth weight, and 1-min APGAR (appearance, pulse, grimace response, activity, and respiration) score (Table 1). These variables are also highly correlated with one another, with high covariates of multicollinearity (variance inflation factor of >10) (Fig. S1). PMA was the most significant factor associated with IP among the four factors (*P* = 0.01; *q* value = 0.015) based on the Hilbert-Schmidt independence criterion (HSIC) (Table S2). Other host factors such as sex and race were not significantly associated with IP. Maternal factors, including preterm premature rupture of membranes (PPROM), maternal antibiotics, antenatal corticosteroids, preeclampsia, and delivery mode, were not associated with IP. These data indicate that younger infants have significantly higher incidences of high IP, likely attributed to their more immature intestinal development.

**TABLE 1:** Study cohort demographics and clinical variables stratified by intestinal permeability category

Variable	Value			*P* value
	Total cohort (*n* = 113)	High IP (*n* = 48)	Low IP (*n* = 65)	
No. (%) of subjects of sex				0.28
Male	61 (54.0)	24 (50.0)	37 (57.0)	
Female	52 (46.0)	24 (50.0)	28 (43.1)	
No. (%) of subjects of race				
White	42 (37.2)	18 (37.5)	24 (37.0)	1.00
African American	63 (55.8)	30 (62.5)	33 (50.8)	0.25
Other	8 (7.1)	0	8 (12.3)	0.02
Mean birth wt (g) ± SD	1,377.8 ± 415.2	1,237.3 ± 378.1	1,496.5 ± 403.0	<0.01
No. (%) of VLBW subjects (<1,500 g)	66 (58.4)	32 (66.7)	34 (52.3)	0.18
Mean gestational age (wks) ± SD	29.8 ± 2.3	29.0 ± 2.3	30.5 ± 2.1	<0.01
No. (%) of early-GA subjects (≤28 wks)	28 (24.8)	18 (37.5)	10 (15.4)	<0.01
Mean postmenstrual age (wks) ± SD	31.1 ± 2.3	30.3 ± 2.3	31.7 ± 2.1	<0.01
No. (%) of early-PMA subjects (<31 wks)	41 (36.3)	23 (47.9)	18 (27.7)	0.03
No. (%) of subjects with caesarean delivery	77 (68.1)	37 (77.1)	40 (61.5)	0.10
No. (%) of mothers with PPROM	36 (31.9)	15 (31.3)	21 (32.3)	1.00
No. (%) of mothers with preeclampsia	25 (22.1)	11 (23.0)	14 (21.5)	1.00
No. (%) of mothers receiving antenatal corticosteroids	106 (94.0)	46 (96.0)	60 (92.3)	0.70
No. (%) of mothers receiving antibiotics	69 (61.1)	30 (62.5)	39 (60.0)	0.85
Mean APGAR score at 1 min ± SD	5.8 ± 2.5	5.3 ±2.8	6.2 ± 2.1	0.04
Mean APGAR score at 5 min ± SD	7.7 ± 1.6	7.5 ± 1.9	7.9 ± 1.6	0.12
No. (%) of subjects receiving antibiotic				
Ampicillin	64 (56.7)	30 (62.5)	34 (52.3)	0.33
Gentamicin	56 (49.6)	25 (52.1)	31 (47.7)	0.70
Vancomycin	8 (7.1)	6 (12.5)	2 (3.1)	0.07
Cefotaxime	9 (8.0)	6 (12.5)	3 (4.6)	0.16
No. (%) of subject who received at least 1 antibiotic vs no antibiotics^*a*^	68 (60.2)	33 (68.8)	35 (53.9)	0.12
No. (%) of subjects who received antibiotic for^*a*^:				
≤3 days	83 (73.5)	30 (62.5)	53 (81.5)	0.03
>3 days	30 (26.6)	18 (37.5)	12 (18.5)	
No. (%) of subjects who received MOM for^*a*^:				
<4 days	26 (23.0)	20 (41.7)	6 (9.2)	<0.01
≥4 days	87 (77.0)	28 (58.3)	59 (90.8)	
Mean feeding duration (no. of days) ± SD^*a*^				
MOM	4.8 ± 2.3	4 ± 2.7	5.5 ± 1.5	<0.01
Formula	1.3 ± 2.3	2 ± 2.7	0.8 ± 1.6	0.02
Mean feeding intake vol received ± SD^*a*^				
MOM	200.8 ± 178.8	123.4 ± 154.2	263.0 ± 175.6	<0.01
Formula	61.7 ± 146.7	99.8 ± 194.7	32.8 ± 91.2	0.03

a Variable measured during the time period starting from the enrollment day (within 1 to 4 days after birth depending on clinical stability) until the day when IP was measured (day 8 ± 2 after birth).

10.1128/mbio.01299-22.8 TABLE S2 Dependence of demographic, obstetric, and neonatal characteristics on intestinal permeability (IP) using the Hilbert-Schmidt independence criterion (HSIC) implemented in the R package dHSIC. Download Table S2, DOCX file, 0.02 MB. Copyright © 2022 Ma et al. 2022 Ma et al. https://creativecommons.org/licenses/by/4.0/ This content is distributed under the terms of the Creative Commons Attribution 4.0 International license.

However, host factors could only partially explain IP. Longer feeding and a higher intake volume of the mother’s own breastmilk (MOM) and a shorter antibiotic treatment duration were also significantly associated with low IP (Table 1). Compared to infants with low IP, neonates with high IP had fewer days of MOM feeding (4 days versus 5.5 days [*P* < 0.01]), a lower total MOM volume (123.4 mL/kg of body weight versus 263 mL/kg [*P* < 0.01]), as well as a longer duration (>3 days) of antibiotic use (37.5% versus 18.5% [*P* = 0.03]). We adjusted host factors associated with IP and fit a generalized logistic regression model. Newborns who were fed MOM for ≥4 days during the first week were demonstrated to be 10.3-fold more likely to have low IP than those who were fed MOM for <4 days (adjusted odds ratio [aOR], 10.3 [95% confidence interval {CI}, 3.21 to 33.33]) (Table 2). Additionally, newborns who had longer antibiotic treatment (≥3 days) were 2.6 times more likely to have high IP; however, this association was mitigated when adjusting for confounders like PMA. This result is in line with our previous observations that antibiotic use is significantly more common in the early-GA subjects (92% for <28 weeks GA versus 32% for >28 weeks GA [*P* < 0.001]) (16). Statistical dependence analyses showed that the cumulative intake volume of MOM prior to the IP measurement was the most significant factor associated with IP (*P* < 0.001; *q* value < 0.01; HSIC statistics = 1.53 and 1.46), at a significance level even higher than those for host factors, including GA (*P* < 0.001; *q* value < 0.01; HSIC statistic = 1.12), PMA (*P* = 0.01; *q* value = 0.015; HSIC statistic = 0.93), and body weight (*P* = 0.01; *q* value = 0.035; HSIC statistic = 1.12) (Table S2).

**TABLE 2:** Odds ratios for factors associated with low IP adjusted for postmenstrual age and birth weight^*a*^

Factor	OR	95% CI for OR	*P* value for OR^*d*^	Adjusted OR^*c*^	95% CI for adjusted OR	*P* value for adjusted OR^*d*^
Duration of antibiotic use^*b*^						
≤3 days	2.65	1.12, 6.25	0.02	1.56	0.58, 4.16	0.37
>3 days	1.0 (ref)			1.0 (ref)		

Duration of MOM feeding^*b*^						
≥4 days	7.04	2.5, 19.6	<0.01	10.30	3.21, 33.33	<0.01
<4 days	1.0 (ref)			1.0 (ref)		

a Fisher’s exact test was used to calculate *P* values for categorical variables. Student’s *t* test was used for continuous variables (birth weight, gestational age [GA], postmenstrual age [PMA], and APGAR scores at 1 min and 5 min). Intestinal permeability (IP) was calculated as the ratio of urine lactulose (La) to rhamnose (Rh), and an La/Rh ratio of <0.05 was defined as low IP. MOM, mother’s own breastmilk; OR, odds ratio; CI, confidence interval.

b Variable measured during the time period starting from the enrollment day (within 1 to 4 days after birth depending on clinical stability) until the day when IP was measured (day 8 ± 2 after birth).

c The adjusted OR model includes PMA and birth weight.

d *P* value calculated using logistic regression.

### Breastmilk intake is associated with improved intestinal barrier integrity.

**FIG 2:**
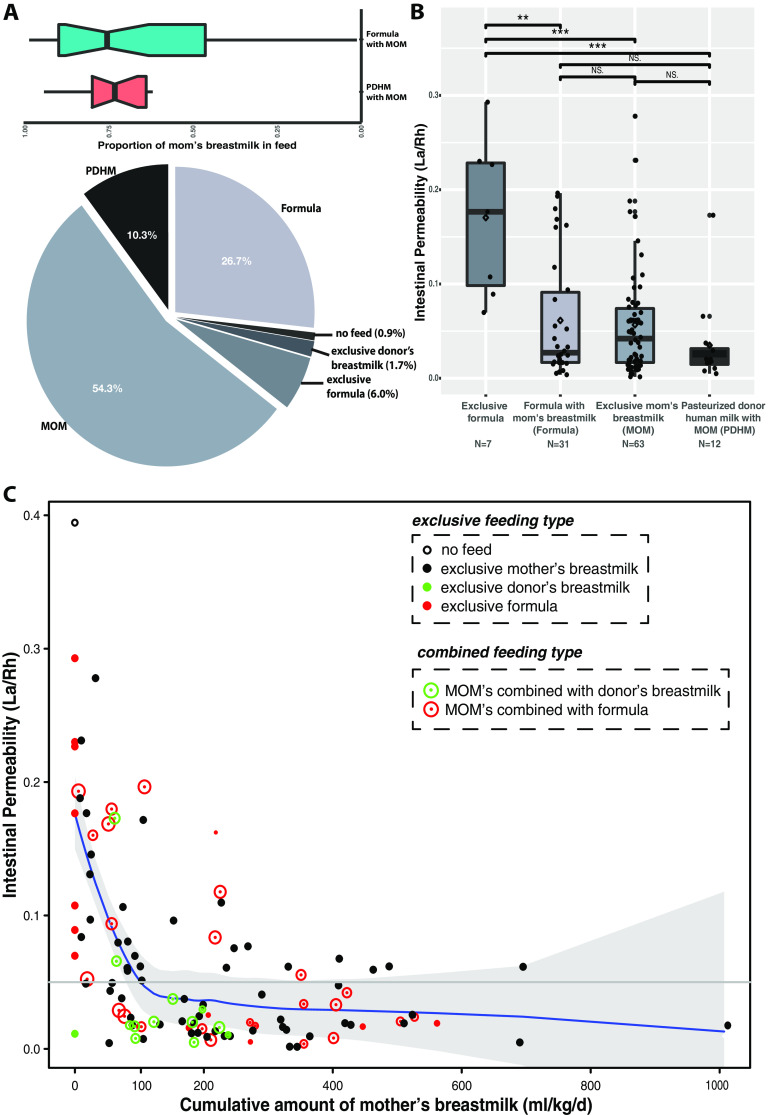
(A) Pie chart of feeding types for the preterm infant population in this study. Abbreviations: MOM, mother’s own breastmilk; PHDM, pasteurized human donor’s milk. (B) Box plot of IP measurements grouped by feeding types. (C) Correlation between intestinal permeability and the cumulative amount of mother’s own breastmilk feeding (milliliters per kilogram) for a total of 113 enrolled preterm infants at 24^0/7^ to 32^6/7^ weeks of gestation. IP was calculated using the ratio of urine lactulose (La) to rhamnose (Rh), and low or high IP was defined by an La/Rh ratio of >0.05 or ≤0.05, respectively. The total amount of mother’s own breastmilk feeding was calculated as the sum of the daily amount of milk intake per kilogram of body weight until days 7 to 10, when IP was measured. Initial feeding was calculated based on 10 mL/kg expressed breastmilk between the first and fourth days of life depending on clinical stability. After 3 to 5 days of initial feeds, feedings were advanced by 20 mL/kg/day until 100 mL/kg/day was reached. Plotted are interquartile ranges (IQRs) (boxes), medians (lines in boxes), and means (diamonds). Significance values were calculated using a Wilcoxon rank sum test. * denotes the level of significance. NS, nonsignificant.

Unfortunately, mothers who deliver preterm often produce less milk than those who deliver at term, and milk administration is often delayed, especially in early preterm infants (27). Formula and/or pasteurized donor human breastmilk (PDHM) is often a necessary dietary supplement. Only 55.7% of neonates in the cohort were exclusively breastfed (*n* = 63); others had their diet complemented with either formula (*n* = 31) or PDHM (*n* = 12) or were fed exclusively formula (*n* = 9) (Fig. 2A). For this reason, we investigated IP in neonates grouped by feeding type. Exclusive formula feeding was significantly associated with high IP, in either the number of days (*P* = 0.02) or the intake volume (*P* = 0.03) (Table 1). However, when formula was used in combination with MOM, even at a minor portion (35.2% ± 31.7% [mean ± SE]), IP was significantly decreased to a level that was no different from that of the cohort fed exclusively MOM (Fig. 2B). Infants whose diet was supplemented with PDHM in addition to MOM had IP similar to that of the group fed exclusively MOM. We further investigated how much MOM is “sufficient” relating to improved IP during the first week after birth. A highly elevated IP was observed in infants who received no MOM (exclusively formula or no feed), and a rapid decrease in IP was inversely correlated with an increased MOM intake volume (Fig. 2C). Discriminatory machine learning schemes suggested that a threshold of around 150 to 180 mL/kg of cumulative intake of MOM by 7 to 10 days of age is associated with low IP. Together, our results indicate that sufficient MOM, used alone or combined with other forms of feeding, significantly impacts IP in early preterm infants. Even more importantly, these results imply that the benefits of breastmilk feeding are beyond nutrition alone but extend to postnatal intestinal barrier maturation.

### Increased *Bifidobacterium* species abundance correlates with improved intestinal barrier integrity.

We further performed high-resolution characterization of the intestinal microbiota in 517 fecal samples, using both short-read sequencing of the V3-V4 region of the 16S rRNA gene on an Illumina HiSeq 2500 instrument (300 bp paired-end reads) (*n* = 472) and long-read sequencing of the full-length 16S rRNA gene on the Pacific Biosciences (PacBio) Sequel II platform (*n* = 192). For short-read sequencing, we obtained a total of 25,838,078 high-quality, nonchimeric ASVs (amplicon sequence variants) after the assembly of forward and reverse reads and quality assessment, representing 51,165 ± 620 (mean ± SE) ASVs per sample (see Table B at https://doi.org/10.6084/m9.figshare.19723252.v1). On the other hand, long-read sequencing generated using circular consensus sequences (CCSs) yielded 1,271,873 high-quality full-length 16S rRNA sequences or 992.9 ± 16.8 (mean ± SE) nonchimeric ASVs per sample. The full-length 16S rRNA gene sequences (1,462 bp on average) extended the partial V3-V4 region (428 bp on average) 3.2 times and afforded species-level assignment for 87.6% of the long-read ASVs (the remaining ASVs were not assigned due to the lack of a reference), compared to 15.3% for the short-read ones (Fig. S2; see also Table D at https://doi.org/10.6084/m9.figshare.19723252.v1). Using samples sequenced by both methods, taxonomic assignments for long-read ASVs were conveyed to short-read ASVs using perfect sequence matches, thus achieving species-level assignment for 65.3% of the short-read sequences (see Table E at https://doi.org/10.6084/m9.figshare.19723252.v1).

10.1128/mbio.01299-22.2 FIG S2 Phylogenetic tree of all ASVs of full-length 16S rRNA genes sequenced on the Pacific Biosciences Sequel II platform. The raxml package (v8.0.0) (1) was used to construct the phylogeny, and the Phyloseq R package (2) was used to display the phylogeny. Download FIG S2, PDF file, 0.2 MB. Copyright © 2022 Ma et al. 2022 Ma et al. https://creativecommons.org/licenses/by/4.0/ This content is distributed under the terms of the Creative Commons Attribution 4.0 International license.

**FIG 3:**
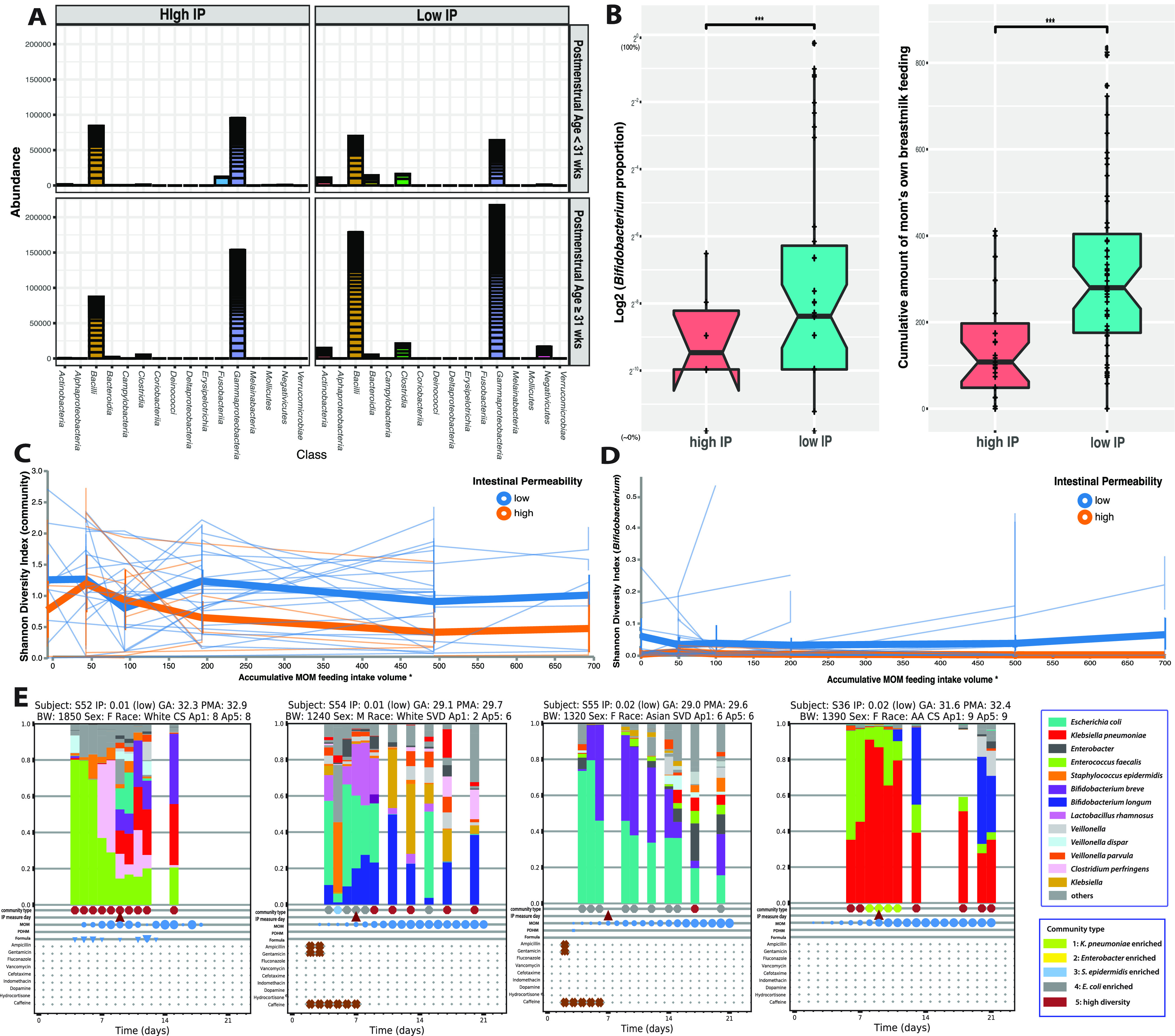
Microbial biomarkers and breastmilk feeding in early preterm subjects with high and low IP. (A) Abundance of bacterial groups stratified by postmenstrual age at study days 7 to 10. The results indicate that the *Actinobacteria* (*Bifidobacterium*) and *Clostridia* (*Clostridiales*) were observed mainly in low-IP subjects but not in high-IP subjects (red). The abundance values of read counts for each ASVs are stacked in order from highest to lowest, separated by a horizontal line. (B) Box plot of *Bifidobacterium* relative abundance and the cumulative amount of mother’s breastmilk feeding (milliliters per kilogram) during the first 7 to 10 postnatal days in subjects with high or low IP. IP was calculated using the ratio of urine lactulose (La) to rhamnose (Rh), with low or high IP defined by an La/Rh ratio of >0.05 or ≤0.05, respectively. Plotted are interquartile ranges (IQRs) (boxes), medians (lines in boxes), and means (diamonds). Significance values were calculated using a Wilcoxon rank sum test. * denotes the level of significance. NS, nonsignificant. (C and D) Volatility plots demonstrating the fluctuation of microbial community diversity (characterized by the Shannon diversity index) (C) and *Bifidobacterium* diversity over mother’s own breastmilk (MOM) feeding volumes in high- or low-IP groups (D). The plot was generated in QIIME (October 2019 version) (106). Nonoverlapping vertical error bars at each measuring point were considered significantly different. (E) Temporal characterization of the intestinal microbiota of early preterm infants with profile changes over the first 21 days after birth. The taxonomic profile was generated using 16S rRNA gene sequencing. Community type is shown in heatmap clusters in Fig. S3 in the supplemental material. The dates when IP was measured, MOM, pasteurized human donor’s milk (PHDM), formula feeding day, and antibiotic administration are shown in the plots. Each circle is sized proportionally to the feeding volume. Abbreviations: BW, body weight; F, female; M, male; CS, cesarean section; SVD, spontaneous vaginal delivery; AA, African American; Ap1, Apgar score 1 minute category; Ap5, Apgar score 5 mintues category; GA, gestational age; PMA, postmenstrual age; BW, birthweight.

In total, 508 ASVs belonging to 212 species in 15 orders and 6 phyla were identified (see Tables A to C at https://doi.org/10.6084/m9.figshare.19723252.v1). The four most abundant taxa were Klebsiella pneumoniae, Escherichia coli, Staphylococcus epidermidis, and Enterobacter spp. These taxa were predominant (>50% relative abundance) and dictated four distinct community types (Fig. S3). These four taxa belong to two classes, *Enterobacteria* (K. pneumoniae, E. coli, and Enterobacter spp.) and *Bacilli* (S. epidermidis), and were highly prevalent (present in 86.2 to 94.8% of the samples) in both high- and low-IP subjects (Fig. 3A). They are also known as “first colonizers” of the infant gut (15, 28, 29). Five other taxa, including Enterococcus faecalis, Clostridium perfringens, Proteus mirabilis, Bifidobacterium breve, and Veillonella dispar, were found to contribute to 17.4% of all sequences and were detected in 47.7 to 86.6% of all samples. These obligate and facultative anaerobes were considered the “succession” microorganisms that succeed the first colonizers (15, 30–32). Together, these nine taxa accounted for 76.0% of all sequences in this data set. The remaining sequences were from a diverse array of obligate and facultative anaerobes (Fig. S3, cluster 5).

10.1128/mbio.01299-22.3 FIG S3 Heatmap of the relative abundances of the 50 most abundant intestinal bacterial taxa in samples collected from 113 preterm infants enrolled in the study. The fecal microbiota was characterized by high-throughput sequencing of the V3-V4 variable regions of the 16S rRNA genes. Ward linkage clustering was used to cluster samples based on their Jensen-Shannon distance calculated using the vegan package in R (1). The number of clusters was validated using gap statistics implemented in the cluster package in R (2) by calculating the goodness-of-clustering measure. Download FIG S3, PDF file, 0.1 MB. Copyright © 2022 Ma et al. 2022 Ma et al. https://creativecommons.org/licenses/by/4.0/ This content is distributed under the terms of the Creative Commons Attribution 4.0 International license.

A zero-inflated negative binomial random-effects (ZINBRE) model was applied to investigate microbial biomarkers correlated with IP. B. breve was the taxon that was most significantly associated with low IP (*P* < 0.001) during the first 7 to 10 days after birth (Fig. 3B, Table S3B, and Fig. S4B). The low-IP group had significantly higher levels of B. breve, more *Bifidobacterium* overall, and more MOM. An adaptive spline logistic regression model was used independently to confirm the association of B. breve with IP and MOM (Fig. S4C and D). Other phylotypes associated with MOM or PMA are shown in Table S3. The high-IP-associated ASVs of S. epidermidis, E. coli, and Parabacteroides distasonis were associated with early PMA (Table S3A). Veillonella dispar was revealed to strongly associate with later PMA (*P* < 0.001) but not with IP. S. epidermidis and E. coli were also associated with less MOM during the first week (Table S3C). B. breve was found in 71.7% of samples containing *Bifidobacterium*, followed by B. longum (21.7%). The other *Bifidobacterium* species were either rare or present at very low abundances (<0.1%). Temporal microbiota profiling indicated that *Bifidobacterium* species reached higher abundances (~5 to 20%) after >3 days of MOM (Fig. 3E) (see https://doi.org/10.6084/m9.figshare.19709923.v1). When stratified by major feeding types, *Bifidobacterium* was most abundant in the cohort fed exclusively MOM or MOM supplemented with formula (Fig. S4A). We plotted community diversity against MOM feeding volume as a function of time and observed that low-IP infants had significantly higher microbiota diversity and higher *Bifidobacterium* species diversity when MOM reached >150 mL/kg of cumulative intake within the first week (Fig. 3C and D). It is worth noting that MOM is a critical but not the only contributor to the abundance of *Bifidobacterium*. Fifteen percent of the subjects who received no MOM had >1% *Bifidobacterium*, and 32.5% had a detectable level of *Bifidobacterium* (>0.1%). Overall, this result further supports the importance of achieving the critical threshold of MOM intake and its critical association with low IP.

10.1128/mbio.01299-22.4 FIG S4 Information on bifidobacterial abundance and intestinal permeability (IP). (A) Relative abundances of bifidobacterial groups stratified by feeding type. The Phyloseq R package (v1.38.0) (1) was used to generate the bar plot. (B) Relative abundances of B. breve between the high-IP and low-IP groups. (C and D) Dependence between IP (C) or MOM feeding dose (D) and the log relative abundance of B. breve. An adaptive spline logistic regression model implemented in the spmrf R package (2) was applied to the phylotypes present in at least 15% of all samples. The Bayesian goodness-of-fit *P* value implemented in the R package rstan (3) was used to assess the significance of the association between phylotypes and the investigated factors. Download FIG S4, PDF file, 0.1 MB. Copyright © 2022 Ma et al. 2022 Ma et al. https://creativecommons.org/licenses/by/4.0/ This content is distributed under the terms of the Creative Commons Attribution 4.0 International license.

10.1128/mbio.01299-22.9 TABLE S3 Taxonomic groups significantly associated with postmenstrual age (PMA), intestinal permeability (IP), and mother’s own milk (MOM) feeding volume. Zero-inflated negative binomial random-effects (ZINBRE) models were used to compute the significance level of the association, which accounts for many zeros as well for correlations between samples from the same subject. All phylotypes detected in at least 15% of the samples were modeled using ZINBRE models. PMA, IP, and MOM feeding volume were modeled as continuous values. (A) Taxonomic groups associated with PMA, which was calculated as the day of life after birth plus gestational age. (B) Taxonomic groups associated with IP, measured 7 to 10 days after birth. (C) Taxonomic groups significantly associated with MOM feeding volume. Download Table S3, XLSX file, 0.02 MB. Copyright © 2022 Ma et al. 2022 Ma et al. https://creativecommons.org/licenses/by/4.0/ This content is distributed under the terms of the Creative Commons Attribution 4.0 International license.

### Population dynamics of *Bifidobacterium* species in early postnatal colonization.

**FIG 4:**
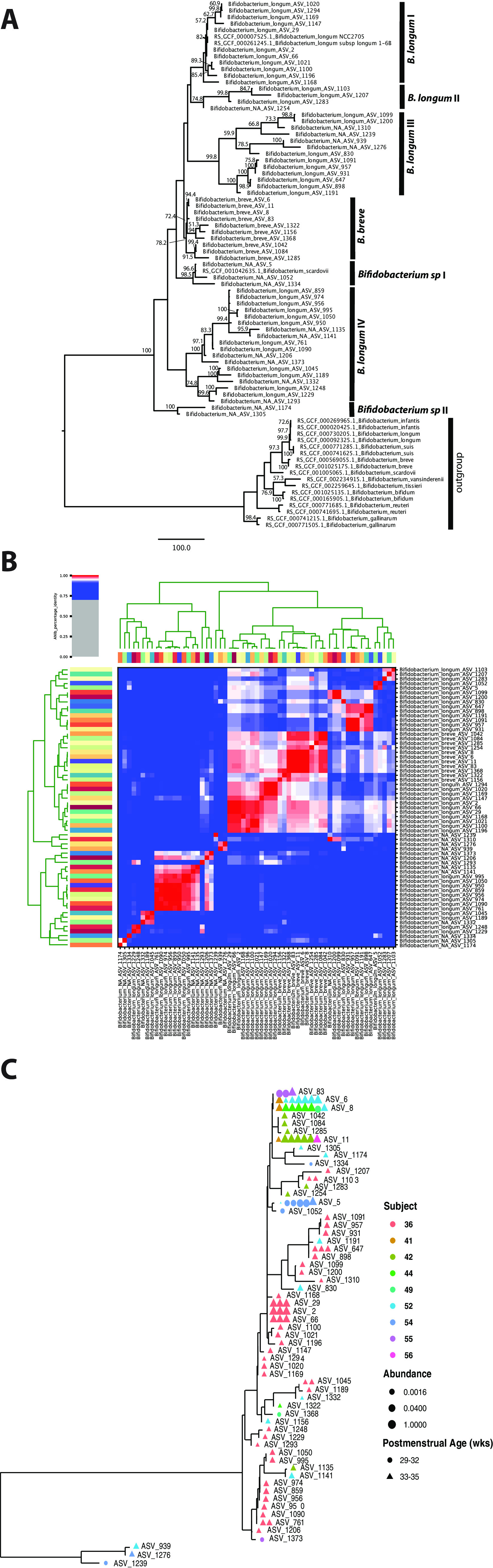
(A) Phylogenetic tree constructed using 81 unique, full-length 16S rRNA gene ASV sequences of *Bifidobacterium*. (B) ANI clustering of full-length 16S rRNA gene sequences. (C) Phylogenetic tree of *Bifidobacterium* ASVs in the stool microbiota of the cohort. All full-length 16S rRNA genes assigned to *Bifidobacterium* were used in the analyses. Color denotes individual subjects.

Phylogenetic analyses of full-length 16S rRNA gene sequences demonstrated that B. breve forms a monophyletic clade, and the four most abundant ASVs were nearly identical, while B. longum was more phylogenetically diverse, with four distinct clades (Fig. 4A and B). Clade I was the most abundant and represented B. longum subsp*. longum*, while B. longum in the other three clades, II to IV, was present at low abundances. ASVs assigned to *Bifidobacterium* showed high sequence diversity (Fig. 4A) as well as inter- and intrasubject variability (Fig. 4C), in that multiple ASVs can be detected in the same subject and a single ASV can be detected in multiple subjects at multiple time points. For instance, 35 B. longum ASVs of four different clades were observed in one subject. Furthermore, some ASVs (i.e., unclassified *Bifidobacterium* spp.) were observed only in infants with an early PMA (<33 weeks), while others did not vary in abundance across PMA (i.e., B. breve), supporting high subspecies-level diversity and population dynamics in the preterm infant gut community.

To characterize the genome content of *Bifidobacterium* species, we performed whole metagenomic sequencing of 30 samples with >10% *Bifidobacterium* species using an Illumina NovaSeq 6000 platform (see Table A at https://doi.org/10.6084/m9.figshare.19723255.v1) and generated 26 B. breve and 4 B. longum nearly complete metagenome-assembled genomes (MAGs) (see Table B at https://doi.org/10.6084/m9.figshare.19723255.v1). We further performed metagenomic sequencing of two samples using the Pacific Biosciences Sequel II platform, which afforded one closed and one nearly complete genome of B. breve strains. The closed genome was 2.34 M in size (Fig. S6; see also Table C at https://doi.org/10.6084/m9.figshare.19723255.v1), similar to the median B. breve genome size of 2.33 M (million bp) in the NCBI database. For pangenome analysis, we supplemented the 26 B. breve in-house MAGs with 107 published genomes (see Table A at https://doi.org/10.6084/m9.figshare.19709917.v2) and the 4 B. longum MAGs with 310 published genomes (see Table B at https://doi.org/10.6084/m9.figshare.19709917.v2) to identify homologous gene clusters (HGCs) (see Tables C and D at https://doi.org/10.6084/m9.figshare.19709917.v2). Among the total of 4,922 B. breve HGCs, 54.2% were considered dispensable (present in <10% of the genomes), 29.4% were core (present in >95% of the genomes), and the rest were accessory (see Table E at https://doi.org/10.6084/m9.figshare.19709917.v2). The pangenome of B. longum (7,265 HGCs) was roughly twice the size of that of B. breve (3,363 HCGs), although the core genomes of the two species were similar (1,511 versus 1,448 HCGs). The large pangenome size of B. longum may reflect its broader host range, which includes both infant and adult intestines, than that of B. breve or B. infantis, which were observed exclusively in the infant gut (33). In particular, the genes involved in the fructose 6-phosphate phosphoketolase-dependent glycolytic pathway for ATP-efficient carbohydrate catabolism, or the “bifid shunt,” are conserved in both species (https://doi.org/10.6084/m9.figshare.19907113). Furthermore, B. longum’s dispensable genome, which comprised 46.3% of its pangenome (2,666 HGCs), was smaller than that of B. breve (54.2%; 3,363 HCGs) in both size and proportion, indicating high genome plasticity in B. breve.

10.1128/mbio.01299-22.5 FIG S5 Metapangenome of Bifidobacterium breve. (A and B) The 26 in-house B. breve MAGs were supplemented with 107 published genomes (https://doi.org/10.6084/m9.figshare.19709917.v2) (A), and our 4 B. longum MAGs were supplemented with 310 published genomes (https://doi.org/10.6084/m9.figshare.19709917.v2) (B) for pangenome construction according to the pangenome workflow (1). The B. breve pangenome is displayed using anvi’o version 6.2 (2). BLASTP was used to compute the average nucleotide identity (ANI) between all pairs of genes. The Markov cluster algorithm (MCL) (3) was used to generate homologous gene clusters (HGCs). Amino acid sequences of each HGC were aligned using MUSCLE (4). HCGs were assigned as core, accessory, or dispensable according to the hierarchical clustering of the gene clusters. Details of each HGC can be found at https://doi.org/10.6084/m9.figshare.19709917.v2. (C) Sourmash version 3.3 (5) was used to compute ANIs across genomes. The source indicates the isolated origin of the genome, and genomes of the same subject are indicated in the same cohort. Download FIG S5, PDF file, 0.4 MB. Copyright © 2022 Ma et al. 2022 Ma et al. https://creativecommons.org/licenses/by/4.0/ This content is distributed under the terms of the Creative Commons Attribution 4.0 International license.

10.1128/mbio.01299-22.6 FIG S6 The complete B. breve genome reconstructed in this study. Metagenomic sequencing of the two selected fecal samples was performed using the Pacific Bioscience Sequel II platform, followed by assembly using Canu v1.8 (1) and deconvolution using BLASTN of the assembly. This complete genome was 2.34 M in size (see https://doi.org/10.6084/m9.figshare.19709923.v1; https://doi.org/10.6084/m9.figshare.19723255), similar to the median B. breve genome size of 2.33 M reported in the NCBI database. (A) KEGG (18 March 2013 release) (2) to characterize the functional categories of B. breve XM1439. (B) Circular genome display of B. breve XM1439, generated by BLAST Ring Image Generator (BRIG) (June 2011 version) (3). (C) Genome alignment of B. breve 1439 and 1437 genomes using MAUVE (4), using B. breve DSM20213 as the reference genome. Download FIG S6, PDF file, 1.1 MB. Copyright © 2022 Ma et al. 2022 Ma et al. https://creativecommons.org/licenses/by/4.0/ This content is distributed under the terms of the Creative Commons Attribution 4.0 International license.

We identified 46 genes specific to B. breve strains colonizing infants with low IP (see Table F at https://doi.org/10.6084/m9.figshare.19709917.v2). While a large number of these genes have unknown functions, others encoded functions such as glycosyl transferases, glycosyl hydrolases, cell surface adhesion and transport, polysaccharide biosynthesis, quorum sensing, and phage integration. Furthermore, a number of functions were significantly enriched in these genomes compared to the publicly available species genomes (adjusted *q* value < 0.05) (see Table F to I at https://doi.org/10.6084/m9.figshare.19709917.v2), such as cation transmembrane transporter activity; glucuronate isomerase; methyladenine glycosylase; glycosyl hydrolase families 59, 2, 85, and 30; and bacterial rhamnosidases A and B. Of note, B. breve HGC profiles appear to be highly similar within subjects, indicating that B. breve genomes detected at different time points in the same infants shared greater similarity than did those from different subjects (https://doi.org/10.6084/m9.figshare.19907113) (see Table J at https://doi.org/10.6084/m9.figshare.19709917.v2). Together, compared to B. longum, B. breve strains colonizing infants with low IP have high genome plasticity and are enriched in genetic features of carbohydrate metabolism and transport that underlie the strong niche-adaptive capabilities of the species.

### Specialized human milk oligosaccharide assimilation capabilities of *Bifidobacterium* strains in early preterm infants.

As both *Bifidobacterium* species abundance and MOM were associated with postnatal intestinal barrier maturation, we next investigated whether these two factors were linked through the ability of *Bifidobacterium* species to utilize the oligosaccharides present in breastmilk. Previously characterized major HMO utilizers like *Bacteroides* species and *Lactobacillus* (34, 35) were largely absent from our cohort (see https://doi.org/10.6084/m9.figshare.19723252.v1), indicating that *Bifidobacterium* species likely provide the genetic capabilities to metabolize HMOs. We thus examined the set of genes encoding extracellular hydrolases, sugar transporters, and intracellular hydrolases (Table S4), which comprise the machinery necessary to take up and metabolize HMO substrates to feed central fermentative metabolism (36–38).

10.1128/mbio.01299-22.10 TABLE S4 *Bifidobacterium* homologous gene clusters (HGCs) characterized as being involved in human milk oligosaccharide assimilation. Genomes were annotated through annotative evidence from the nomenclature of the Consortium for Function Glycomics, eggNOG (v4.5) (1), KEGG (18 March 2013 FTP release) (2), Pfam (v30.0) (3), and CAZy (2014 release) (4, 5). Similarity searches were performed according to previously annotated enzymes or transporter proteins based on the accession numbers listed in previous studies (6–8) using a BLASTP similarity search and confirmed with COG, Pfam, and CAZy annotation evidence to ensure the integrity of the results. (A) HGCs involved with extracellular enzymes and their homologs involved in the extracellular cleavage of HMOs. (B) HGCs characterized as family 1 solute binding proteins (F1SBP). (C) HGCs involved with enzymes for catabolizing HMO substrates intracellularly. (D) HGCs characterized as FHMO (fucosylated human milk oligosaccharide utilization cluster). (E) HGCs involved in sialylated HMO substrate catabolism. (F) HGCs involved in sulfatase catabolism activity. Download Table S4, XLSX file, 0.03 MB. Copyright © 2022 Ma et al. 2022 Ma et al. https://creativecommons.org/licenses/by/4.0/ This content is distributed under the terms of the Creative Commons Attribution 4.0 International license.

Intracellular HMO utilization functions were found to be encoded exclusively by both B. breve and B. longum. We examined eight essential extracellular enzymes and their homologs (for details, see Materials and Methods) known to be required for the extracellular breakdown of HMOs into smaller molecules that are then transported intracellularly. Interestingly, none of these extracellular enzymes were found in this cohort. We investigated five essential bacterial ABC transporters and homologs involved in the import of various oligosaccharides, known to have a high specificity for HMOs conferred by substrate binding protein (SBP) domains (39). Both B. breve and B. longum contained *gltA* (Table S4A), a gene considered crucial for the import of lacto-*N*-tetraose (LNT). LNT comprises the core HMO structure that is catabolized via lacto-*N*-biose (LNB) intermediates (40). Furthermore, a family 1 solute binding protein (F1SBP) gene cluster, Blon_2177, was found in both B. breve and B. longum (Table S4B). This cluster was found to be critical for the import of nonfucosylated type 1 oligosaccharides (41). None of the B. longum strains but the majority of the B. breve strains of this cohort (92.4%) harbor the LNnT (lacto-*N*-neotetraose) transporter that is encoded by *nahS*. These findings indicate that both B. breve and B. longum could transport LNB and LNT, while B. breve can further metabolize LNnT.

We then evaluated the capability of consuming the transported oligosaccharides, and compared to B. longum, we revealed expanded metabolic capabilities of B. breve strains of this cohort to utilize a variety of HMO molecules, including fucosylated or sialylated forms, in addition to the neutral types of HMOs (i.e., LNB, LNT, and LNnT). Seventeen key glycoside hydrolases (GHs) involved in essential HMO degradation and utilization were investigated (Table S4C). The key intracellular enzymes GH2 (β-1,4-galactosidases) (LacZ2/6), GH112 (galacto-*N*-biose [GNB]/LNB phosphorylase) (*lnpA*), GH20 (β-*N*-acetylglucosaminidase), and GH42 (β-1,3-galactosidase) (*lntA*; bga42A) are highly conserved in both B. breve and B. longum. These enzymes lack transmembrane domains or signal peptide sequences and are required to degrade HMOs intracellularly (42). While almost all B. breve strains contained GH95 α-fucosidase (*afcA*) (homolog of Blon_2335), GH33 α-sialidase (homolog of Blon_0646), and GH20 β-*N*-acetylglucosaminidase (*nahA*) (homolog of Blon_0459) (Table S4C), only a small portion of B. longum strains (~10%) contained these enzymes. Furthermore, B. breve strains present in these preterm infants carry the gene encoding GH29 α-fucosidases more often (53.8% versus 12.7%) than B. breve strains isolated from other sources obtained from GenBank. The presence of GH29 α-fucosidase genes underlines the ability to consume fucosylated oligosaccharides such as 2′-fucosyllactose (2′-FL) and larger fucosylated HMOs such as lacto-*N*-fucopentaose (38, 42). The GH29-containing B. breve strains in our cohort also encode GH95. In fact, GH29 and GH95 α-fucosidases are highly complementary since they target specific substrates of α-1,3/4- and α-1,2-fucosyl linkages, respectively (42), and the activation of both enzymes enables the degradation and utilization of a larger variety of HMOs. Moreover, a prominent gene cluster termed FHMO (fucosylated human milk oligosaccharide) that contains both GH29 and GH95 α-fucosidase-encoding genes was observed in some B. breve strains but was largely absent from B. longum strains (Table S4D). This cluster was reported to enable B. breve strains to preferentially consume fucosylated HMOs over neutral HMOs during early bacterial growth (42). In particular, the putative fucosyllactose SBP (BLNG_1257) present in this cluster confers glycan binding specificity and is present consistently in B. breve strains of this cohort but rarely in other B. breve strains in GenBank. Overall, our results revealed an expanded, specialized HMO assimilation capability of B. breve strains, conferring a competitive growth advantage in the gut of this preterm infant cohort when fed breastmilk.

### Host-derived glycoprotein utilization is limited to B. breve in early preterm infants.

Besides HMOs, host-derived glycoproteins such as mucin and proteoglycan (mucus or milk) are critical carbon sources for bacteria in the infant intestinal microenvironment. Human glycoproteins are often heavily sulfated and could not be metabolized without bacterial glycosidases (43, 44). We investigated two sulfatase-encoding gene clusters essential for sulfatase metabolism, *ats1* and *ats2* (45, 46), and they each encode glycosulfatases and the accompanying anaerobic sulfatase-maturing enzymes (anSMEs) with an associated transport system and transcriptional regulator (46). The primary mucin degradation capabilities of this cohort are shown to be limited to B. breve strains (Table S4F), as the two clusters are present in 100% of B. breve strains in our cohort and ~70% of all B. breve genomes available. B. longum strains rarely harbor *ats1*, and no strains carry *ats2*.

In addition to sulfated residues, more than half of human colonic mucin oligosaccharides also contain sialic acid residues (47). The release of sialic acid is an initial step in the sequential degradation of mucins and sialylated HMO substrates (46, 48). Hence, we investigated the two gene clusters essential for the uptake and metabolism of sialic acid, the *nagA2-nagB3* cluster (Bbr_1247-Bbr_1248) and the *nan-nag* cluster (Bbr_0160-Bbr_0172) (49–51). These two gene clusters are highly conserved in B. breve, while they are present in only 14% of B. longum genomes (https://doi.org/10.6084/m9.figshare.19709917). Our results demonstrate that the capability of foraging sulfated and/or sialylated host-derived glycoproteins is attributed to B. breve strains in this cohort. This metabolic versatility of B. breve may greatly improve its fitness and facilitate its mucosal adherence, hence facilitating colonization under nutrient- or energy-limited conditions in the preterm infant gut environment.

## DISCUSSION

Early preterm neonates are a vulnerable and challenging population that often requires intensive medical care. As a result of their premature birth, these neonates often have an aberrantly permeable intestinal barrier that fails to limit bacterial translocation. Our group has previously reported positive associations between persistently elevated intestinal permeability and delayed feeding, prolonged antibiotic exposure, and altered development of the intestinal microbiota as well as a lack of a progressively increased abundance of *Clostridiales* (15, 16). These *Clostridiales* became abundant mostly at the end of the second week after birth; this is after the extensive barrier maturation that occurs during the first week. In this study, we determined the minimal intake of maternal breastmilk necessary to significantly decrease IP and identified specific *Bifidobacterium* species and strains as biomarkers associated with low-IP development in preterm infants in the first week of life.

**FIG 5:**
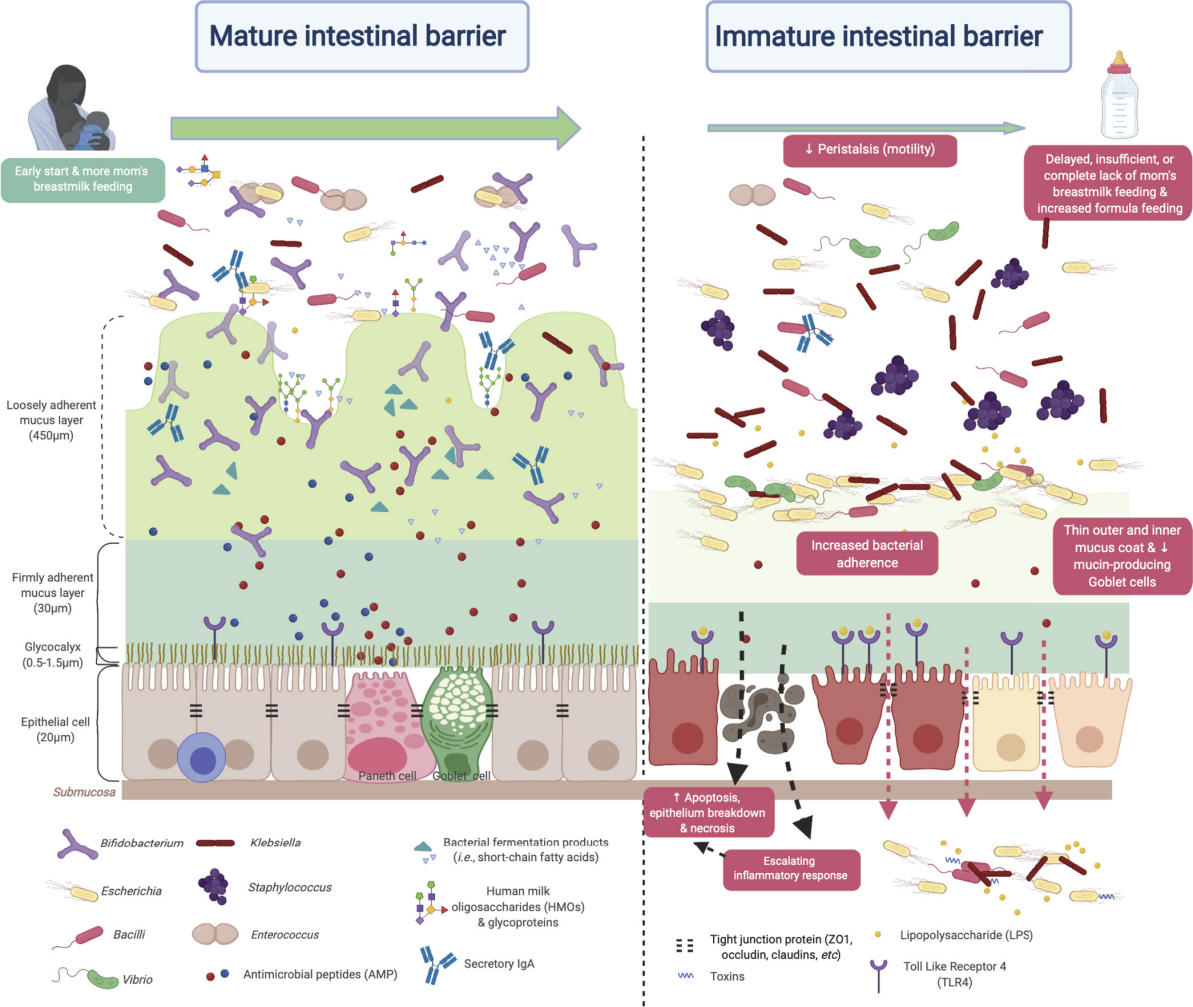
Illustration of mature and immature intestinal barriers in neonates. Peristalsis (reduced intestinal motility), maldigestion of nutrient sources, and a compromised gut barrier may render the mucosa susceptible to invasion by opportunistic pathogens in the gut environment. The resulting imbalance between epithelial cell injury and repair leads to a vicious cycle of maldigestion, bacterial invasion, immune activation, and uncontrolled inflammation. The illustration is not drawn to scale. (Created with BioRender.com.)

We posited that the benefits of breastmilk extend beyond nutrition and include improved gut barrier function and that the two factors associated with reduced IP, MOM feeding and *Bifidobacterium* strains, are, at least in part, linked by the ability of *Bifidobacterium* to metabolize human milk oligosaccharides (illustrated in Fig. 5). To investigate this link, we evaluated the carbohydrate-metabolizing capabilities of *Bifidobacterium* strains and uncovered a complement of genes dedicated to utilizing a wide variety of HMO molecules as well as host-derived glycoproteins. These genetic features were enriched in preterm infant gut-associated *Bifidobacterium* strains compared to those isolated from other sources like dairies or the adult gut. Our results are concordant with those of previous studies showing that the establishment of a bifidobacterium-dominant community was facilitated by specific gene clusters supporting HMO metabolism, which are absent in many adult-associated bifidobacterial strains (52–55). Functional characterization of the contribution of B. breve metabolizing MOM to low IP would be critical for its translational significance. Future studies modeling both the transcriptional activities of bifidobacterial biomarkers and host responses in a longitudinal design are warranted to address the cause-effect relationships of MOM and *Bifidobacterium* for intestinal barrier maturation. Furthermore, the production of short-chain fatty acids via carbohydrate consumption by bifidobacteria, particularly acetate and butyrate, was demonstrated to correlate with their anti-inflammatory properties and promoted the defense functions of the epithelium (56–58). Together, the results of our study support the notion that intestinal barrier function can develop postnatally, and this process could be induced through supplementation with breastmilk substrates as well as *Bifidobacterium* strains that consume them. These elements are promising therapeutic targets to reduce NEC and other life-threatening conditions associated with intestinal hyperpermeability.

B. breve is a known dominant *Bifidobacterium* species in both preterm and term infant gut microbiota (59) and was also observed in breastmilk and vaginal microbiota (60, 61). In humans, B. breve appears to be found exclusively in these environments and is largely absent in the adult gut. The factors contributing to B. breve persistence in infants are not well understood. Most studies were performed using the type strain B. breve ATCC 15700 (JCM1192), which has a limited ability to consume HMOs (62, 63). As demonstrated by us and others, strains of B. breve vary greatly in their abilities to metabolize HMOs (55). The B. breve strains in our cohort displayed extensive enzymatic capabilities designed to efficiently utilize a broad range of dietary and host-derived carbohydrates, thus maximizing their colonization of the infant intestinal environment. In particular, we demonstrated that LNnT utilization was limited exclusively to strains of B. breve. Growth on LNnT was shown *in vitro* to enable *B. infantis* to outcompete other species such as *Bacteroides* species (64). LNnT can be fermented by specific strains of *Bifidobacterium* found only in the infant gut (65). Digestion of neutral HMOs (i.e., LNT and LNnT) was actually shown to induce a significant shift in the ratio of secreted acetate to lactate compared to the catabolism of the simpler carbohydrates that they contain (66). Furthermore, the GH29 α-fucosidase, an uncommon enzyme correlated with the ability to grow on fucosylated HMOs (38), was enriched in only B. breve strains in this cohort. The presence of key gene sets expands B. breve metabolic capabilities (i.e., FHMO, GH29, and GH95) and is reminiscent of those found in *B. infantis* ATCC 15697, the model strain that can also consume a broad repertoire of HMOs (41, 67). Previous clinical trials administering B. breve strains in early preterm infants yielded contradicting results, which may be related to the selection of different strains. For example, Kitajima and coauthors reported that a B. breve BBG strain could colonize the immature bowel effectively, with significantly fewer abnormal abdominal signs and greater weight gain in VLBW infants (68). However, the clinical trial of the type strain BBG-001 in very-preterm infants observed no evidence of a benefit in terms of preventing NEC and late-onset sepsis (LOS) (69). These data highlight the importance of strain characterization in prophylactic supplementation with live biotherapeutics. Further characterization of these key genes will be necessary to understand the range of oligosaccharides that B. breve strains can transport and consume. Collections of B. breve strains isolated from both preterm infants with rapidly decreasing IP and healthy term infants should be established to achieve this important goal.

The specialized HMO and glycoprotein utilization capabilities of B. breve, particularly the degradation of sulfated and sialic residues, further confer a competitive capability that improves B. breve fitness and facilitates its adherence to and colonization of the gut mucosa (70). The release of sialic acid is an initial step in the sequential degradation of mucins and sialylated HMO substrates (46, 48), and the abilities to utilize the heavily sulfated mucin glycoprotein and sialic residues were found to be highly correlated (46, 49). Sialic acid concentrations are highest in the colostrum in preterm infants but decrease by almost 80% after 3 months (71). Furthermore, breastmilk from mothers who delivered preterm was reported to be a rich source of oligosaccharide-bound sialic acids, with 20% more sialic acid residues than in breastmilk from term mothers and 25% more than that found in formula (72). A recent *in vivo* study showed that sialylated HMOs are on the causal pathway of a microbiota-dependent infant growth outcome and hence were considered the most growth-discriminatory HMO structures (73). Interestingly, and supporting its importance in infant health, only strains of *B. infantis* and B. breve isolated from the infant gut have been reported to be capable of utilizing sialic acid and sialylated lacto-*N*-tetraose as sole carbon sources (54, 74, 75). A few B. breve strains were actually reported to preferentially consume sialylated HMOs, in particular sialyl-LNT b (LSTb) and sialyl-lacto-*N*-hexaose (S-LNH), over neutral HMOs (38, 49). Given that bacteria with pathogenic potential are capable of utilizing sialic acid, B. breve strains could rapidly sequester sialic acid away from these pathogens and offer nutritional immunity, i.e., sequestering nutrients to limit infection, thus contributing to a healthy intestinal environment (76). It would be highly insightful to further characterize maternal HMO variations in MOM and the composition of specific formulas, in addition to the information on HMO assimilation capabilities of bifidobacterial strains, for a comprehensive understanding of the essential factors contributing to postnatal intestinal maturation.

HMO utilization by *Bifidobacterium* species in this cohort appears to be exclusively an intracellular process, which would be unlikely to allow cross-feeding of intermediates with other gut bacterial species. Extracellular digestion of HMOs would afford fucose and sialic acid monomers to be cross-fed to other bacteria, some of which have pathogenic properties (77). *Bacteroides* spp., which are largely absent in this cohort, are known to employ an exclusively extracellular process in HMO utilization (64). The “internalize, then degrade” approach for HMO consumption is a critical *Bifidobacterium* property that affords protection against infection for the infants. Interestingly, the preference for intracellular digestion of HMOs is not conserved across all infant gut *Bifidobacterium* species or strains. A recent study revealed that *Bifidobacterium* strains in the gut microbiome of breastfed infants in Malawi and Venezuela similarly employed an intracellular HMO digestion strategy, while *Bifidobacterium* strains in a cohort of U.S. infants fed formula and breastmilk preferentially employed extracellular HMO digestion strategies (36). This difference may relate to galactooligosaccharide (GOS) transporter genes present in strains that internalize HMOs to metabolize them, especially the GNB/LNB-BP (*gltA*) gene (36, 78), although the mechanisms remain unclear.

Our study highlights the strong potential for the prophylactic administration of specific B. breve strains early in life along with specific HMOs to enhance the intestinal barrier in preterm neonates. We previously defined a “window of opportunity” of day 8 ± 2 after birth for intervention prior to the onset of leaky gut-associated conditions such as NEC (15, 16). Our study proposed the role of breastmilk feeding in promoting the growth of beneficial *Bifidobacterium* species and strains that could consume breastmilk HMOs during that critical window period of time. In the absence of these prophylactic *Bifidobacterium* strains, the benefit of breastmilk feeding is expected to be dramatically reduced. Counting on the vertical transmission of these *Bifidobacterium* strains from the mother’s gut or vaginal microbiota or breastmilk is not reliable and could leave many infants unprotected (79, 80). It is thus critical to gain further mechanistic insight into bifidobacterium-rich microbiota formation in the infant gut by prophylactic supplementation with live biotherapeutics that possess the ability to effectively utilize them. Such an understanding will inform the design of clinical interventions with supplementation with HMOs and *Bifidobacterium* as live biotherapeutic prophylaxis to enhance intestinal barrier integrity early in life and ultimately reduce the risk of NEC.

## MATERIALS AND METHODS

### Study cohort and feeding protocol.

The study protocol was approved by the institutional review boards of the University of Maryland, Baltimore, and Mercy Medical Center. Written informed parental consent was obtained. Eligibility criteria were described previously (16). One hundred thirteen eligible preterm infants at 24^0/7^ to 32^6/7^ weeks of gestation were enrolled within 4 days after birth from combining cohorts enrolled from June 2013 to October 2014 and from October 2018 to November 2019. Prior to the study procedures, a complete physical examination, including vital signs, weight, height, and head circumference, was performed. Demographic, obstetric, clinical, medication exposure, feeding practice, and adverse event data were collected from the medical record.

Enteral feeds by the orogastric or nasogastric route were initiated between the first and fourth day of life depending on clinical stability. After initial feeds of 10 mL/kg expressed breastmilk or 20 kcal/oz preterm formula daily for 3 to 5 days, feedings were advanced by 20 mL/kg/day until 100 mL/kg/day was reached. Subsequently, caloric density was advanced to 24 kcal/oz prior to increasing the feeding volume by 20 mL/kg/day to 150 mL/kg/day. The total volume of each source of feeds was calculated as the sum of the daily amount of milk intake per kilogram of the administered expressed mother’s breastmilk, donor milk, or preterm formula from the initial feed day until postnatal days 7 to 10, when intestinal permeability (IP) was measured. Feedings were held or discontinued for signs of feeding intolerance such as abdominal distension, gastric residuals, or hematochezia or for clinical deterioration. Pooled pasteurized human donor breastmilk (PHDB) was purchased from Prolacta Biosciences (Duarte, CA, USA). PHDB was collected from mothers of term infants who have breastfed for at least 6 months (81).

### *In vivo* intestinal permeability measurement.

In our previous pilot studies that employed a small cohort of neonates (*n* = 37) (15, 16) with IP measured at study days 1, 8 ± 2, and 15 ± 2, it was shown that IP is high within 4 days of birth in all preterm infants, with a rapid maturation of the intestinal barrier over the first week of life. A persistently high IP and/or a late increase in IP indicates the physiological immaturity of intestinal tract barrier function. Hence, the first 7 to 10 days in preterm infants are a critical observation period for monitoring IP. Eligible preterm infants received 1 mL/kg of the nonmetabolized sugar probes on postnatal days 7 to 10, which included lactulose (La; Cumberland Pharmaceuticals, Nashville, TN), which is a marker of intestinal paracellular transport, and rhamnose (Rh; Saccharides, Inc., Calgary, Alberta, Canada), which is a marker of intestinal transcellular transport. One milliliter of a solution containing 8.6 g La plus 140 mg Rh/100 mL was administered enterally by nipple or by gavage via a clinically indicated orogastric tube (82). A minimum of 2 mL of urine was collected over a 4-h period following the administration of the La/Rh dose as previously described (16). La and Rh concentrations were measured by high-pressure liquid chromatography (HPLC) at the University of Calgary (Calgary, Canada). High or low intestinal permeability was defined by an La/Rh ratio of >0.05 or ≤0.05, respectively, as validated and applied previously (16). Postmenstrual age at sugar probe dosing was calculated as the gestational age at birth plus the postnatal age on the dosing day (83).

### Fecal specimen collection and nucleic acid extraction.

Fecal samples (~1 g) collected daily from enrollment until postnatal day 21 or NICU discharge were stored immediately in 1 mL of DNA/RNA Shield (Zymo Research, Irving, CA, USA). Stool specimens were collected from within the stool mass from the diaper as much as feasible to avoid frequent air exposure. The stool sitting time was 0 to 3 h, and the sample was collected during diaper changes every 3 h. Urine and fecal samples were archived at −80°C until processing.

Genomic DNA was extracted from homogenized fecal samples using the MagAttract PowerMicrobiome DNA/RNA kit (Qiagen) implemented on a Hamilton Star robotic platform and after a bead-beating step on a TissueLyser II instrument (Qiagen) in 96-deep-well plates at the Microbiome Service Laboratory (MSL) at the University of Maryland, Baltimore (Baltimore, MD, USA). DNA purification from lysates was done on a QIAsymphony automated platform.

### Short-read sequencing of 16S rRNA gene amplicons and whole-community metagenomes.

PCR amplification of the 16S rRNA gene V3-V4 hypervariable region was performed using dual-barcoded universal primers 318F and 806R as previously described (84). In brief, amplicon pools were prepared for sequencing with AMPure XT beads (Beckman Coulter Genomics, Danvers, MA), and the size and quantity of the amplicon library were assessed on the LabChip GX system (PerkinElmer, Waltham, MA) and with a library quantification kit for Illumina (Kapa Biosciences, Woburn, MA), respectively. The PhiX control library (v3) (Illumina, San Diego, CA) was combined with the amplicon library. High-throughput sequencing of the amplicons was performed on an Illumina MiSeq platform using the 300-bp paired-end protocol. Sequence libraries were prepared from the extracted DNA using the Nextera DNA Flex kit (Illumina, San Diego, CA) according to the manufacturer’s specifications. Libraries were then pooled in equimolar proportions and sequenced on a single Illumina NovaSeq 6000 S2 flow cell providing an average of 6.5 million pairs of 150-bp reads per library at the Genomic Resource Center at the University of Maryland School of Medicine.

### Long-read sequencing of the full-length 16S rRNA gene and whole-community metagenomes on the Pacific Biosciences Sequel II platform.

Amplification of the full-length 16S rRNA gene was performed using dual-barcode, two-step PCR on diluted (1:10) genomic DNA. The first round of PCR amplification of the 16S rRNA full-length gene was performed using universal primers 27F (AGRGTTYGATYMTGGCTCAG) and 1492R (RGYTACCTTGTTACGACTT) according to Pacific Biosciences (Menlo Park, CA, USA) specifications for 20 cycles. The cycling conditions for the first-step PCR were 95°C for 30 s, 57°C for 30 s, and 72°C for 60 s. The PCR mixture was then diluted in water (1:5) and amplified with Pacific Biosciences universal forward/reverse 96-plate primers for an additional 20 cycles according to Pacific Biosciences specifications. Cycling conditions are described in the manufacturer’s protocol (85). DNA quantification was carried out using the Quant-iT PicoGreen double-stranded DNA assay (Invitrogen) and visualized on a 2% agarose E-gel. The amplicon libraries were normalized, cleaned, and concentrated using AmPure XP SPRI beads (Beckman Coulter, Brea, CA, USA) at 0.6× the reaction volume.

Library pools were prepared with SMRTBell template prep kit 1.0 with barcoded adaptors. Libraries were then size selected on a BluePippen system (Sage Science, Beverly, MA) with a cutoff of 5 kb. Sequencing was performed on the Sequel II platform (PacBio, Menlo Park, CA) with loading at 60 pM. Multiplexed samples were sequenced on PacBio Sequel II cells using S/P3-C1/5.0-8M sequencing chemistry. Demultiplexing was done with lima (version 1.9.0) using default parameters, except for a minimum barcode score of 26 and a minimum length of 50 bp; both tools are part of the SMRTLink 6.0.1 software package with updated CCS version 3.4.1 (Pacific Biosciences, 2019). Raw reads were assembled via Canu v1.8 and the -pacbio-raw protocol (86). The resulting contigs were taxonomically annotated using BLASTN v2.8.1 (87) and the nonredundant nucleotide database (updated on 3 May 2019) to pool all contigs identified under the same species name to form metagenomic bins. Binned contigs were circularized and rotated using Simple-circularise (88) and were retained if the circularized contigs were in the range of the full genome size according to published closed genomes of that species based on the GenBank genome database. Metagenome bins were further confirmed using GTDB-Tk v1.1.0 (89). Genomes were annotated using PROKKA v1.13 (90).

### Epidemiological analyses.

Covariates identified based on previous literature and biological plausibility were collected at the time of enrollment of the participants and evaluated. Categorical data were compared using Fisher’s exact test, and continuous data were compared using Student’s *t* test. Multicollinearity between covariates was assessed using the variance inflation factor (VIF) and tolerance, where covariates with a VIF of >10 were considered collinear. Covariates with a *P* value of <0.05 in the bivariate analysis were considered confounding factors and were adjusted in the multivariable analysis as random factors. Generalized logistic regression was used to determine the association between IP category and continuous variables, including the duration of antibiotics and the duration of MOM feeding. Analyses were conducted using SAS version 9.4 software (SAS Institute, Cary, NC), and the code used for this statistical analysis was deposited at https://github.com/igsbma/IP_microbiome/tree/main/statistical_analyses.

### Bioinformatics analysis of the intestinal microbiota.

For 16S rRNA V3-V4 gene amplicon analysis, raw data were demultiplexed, and barcode, adaptor, and primer sequences were trimmed using tagcleaner v0.16 (91). Quality assessment and sequencing error correction were performed using the DADA2 v1.14 software package (92) and the following parameters: forward reads were truncated at position 240 and reverse reads were truncated at position 210 based on the sequencing quality plot, and no ambiguous bases and a maximum of 2 expected errors per read were allowed (93). The quality-trimmed reads were used to infer amplicon sequence variants (ASVs) and their relative abundances in each sample after removing chimeras. The SILVA database (94), release 132, was used to assign taxonomy. The following criteria were applied for an ASV: (i) the ASV was at least 400 bp in length for long-read sequencing, (ii) it was observed in at least two samples, (iii) there were at least 5 counts in at least one sample, and (iv) it was not assigned to taxonomic groups of mitochondria or chloroplasts.

For full-length 16S rRNA gene analyses, CCS reads were generated using the ccs application with a minPredictedAccuracy of 0.99, and the rest of the parameters were default, including a minimum of 3 subread passes. Demultiplexing was done with lima (version 1.9.0) with a minimum barcode score of 26 and a minimum length of 50 bp; both tools are part of the SMRTLink 6.0.1 software package with updated CCS version 3.4.1 (Pacific Biosciences, 2019). The microbiota analyses were modified from a previously reported bioinformatics pipeline that incorporates the DADA2 protocol (95). The quality-trimmed reads were used to infer ribosomal sequence variants and their relative abundance in each sample after removing chimeras. Taxonomy was assigned to each ASV generated by DADA2 using both the SILVA (release 132) database and the Genome Taxonomy Database (GTDB) (96) and the RDP naive Bayesian classifier as implemented in the DADA2 R package (97, 98). In a few cases when conflicting taxonomic assignments appeared, the NCBI RefSeq 16S rRNA database combined with the RDP database (99, 100) and the Human Intestinal 16S rRNA database (HITdb v1) (101) was used to resolve the conflict. Pacific Biosciences long-read sequencing complements short-read sequencing for its high accuracy and extended length. To boost taxonomy assignments for short-read sequencing, we performed a BLASTN search of the short-read ASVs to the long-read ASVs and assigned a taxonomic name to the short reads if there was 100% identity and a unanimous assignment if there were multiple hits for long-read sequences.

A heatmap was constructed from the relative abundances of the 50 most abundant intestinal bacterial taxa in samples collected from the 113 preterm infants enrolled in the study. The ASVs were normalized using the total sum to calculate their relative abundances. Ward linkage clustering was used to cluster samples based on their Jensen-Shannon distance calculated using the vegan package in R (102). The number of clusters was validated using gap statistics implemented in the cluster package in R (103) by calculating the goodness-of-clustering measure. The raxml package (v8.0.0) (104) was used to construct the phylogeny, and the Phyloseq R package (v1.38.0) (105) was used to display the phylogeny and the bar plot. A volatility plot was used to demonstrate the fluctuation of microbial community diversity (characterized by the Shannon diversity index) over the MOM feeding volume in the high- or low-IP groups. The plot was generated in QIIME (October 2019 version) (106) (option-longitudinal plot-feature-volatility).

### Statistical analysis of the intestinal microbial community.

The Hilbert-Schmidt independence criterion (HSIC) R package dHSIC (107) was used to examine the independence between any variables and IP. Longitudinal modeling was performed using zero-inflated negative binomial random-effects (ZINBRE) models. These models account for the possibility of the existence of more than the expected zeros (from the negative binomial distribution) as well for correlations between samples from the same subject. Although IP was categorized into high and low groups, it is inherently continuous, and hence, we modeled IP as a continuous value in our analyses. Subject was included as a random factor. Read count data of phylotypes detected in at least 15% of the samples were modeled using ZINBRE models. The same principle was applied to MOM and PMA. The model was fitted using the JAGS R package (108), and 10,000 iterations with the same number of burn-in iterations were used. The convergence of the model was assessed using Gelman and Rubin’s potential scale reduction factor (109) and visual inspection of each coefficient’s Markov chains. The means of the posterior distributions of the estimated coefficients and their corresponding 95% credible intervals were calculated using the model’s Markov chains. The credible intervals without overlap are considered significant. *P* values were computed by assuming the normality of the posterior distributions of the corresponding coefficients. An adaptive spline logistic regression model implemented in the spmrf R package (110) was used independently to confirm the association of B. breve with IP and MOM. This model is a locally adaptive nonparametric fitting method that operates within a Bayesian framework, which uses shrinkage-prior Markov random fields to induce sparsity and provides a combination of local adaptation and global control (110). The Bayesian goodness-of-fit *P* value implemented in the R package rstan (111) was used to assess the significance of the association. The R code implementation of the model has been deposited at https://github.com/igsbma/IP_microbiome/tree/main/statistical_analyses. Discriminatory machine learning scheme computations were implemented in weka (112, 113), including J48 decision tree, REPTree, decision stump, and logistic model trees. The functional enrichment test was performed for each functional group (based on Clusters of Orthologous Groups [COG] and Pfam annotations) and each of the homologous gene clusters (HGCs) generated in genome comparison analyses. Frequency tables of each function or HGC in each category (i.e., metagenome-assembled genomes [MAGs] from this study versus the GenBank genomes) were generated, which were used to fit a generalized linear model with the logit linkage function to compute an enrichment score and *P* value for each unit (114). False detection rate correction for *P* values was used to account for multiple tests using the R package qvalue (115).

### Intestinal microbiome analyses.

Metagenomic sequence data were preprocessed using the following steps: (i) human sequence reads and rRNA large-subunit (LSU)/small-subunit (SSU) reads were removed using BMTagger v3.101 (116) using a standard human genome reference (GRCh37.p5) (117); (ii) rRNA sequence reads were removed *in silico* by aligning all reads using Bowtie v1 (118) to the SILVA PARC ribosomal-subunit sequence database (94), and sequence read pairs were removed even if only one of the reads matched the human genome reference or rRNA; (iii) the Illumina adaptor was trimmed using Trimmomatic (119); (iv) sequence reads with an average quality greater than *Q*_15_ over a sliding window of 4 bp were trimmed before the window, assessed for length, and removed if they were <75% of the original length; and (v) no ambiguous base pairs were allowed. The taxonomic composition of the microbiomes was established using MetaPhlAn version 2 (120). The MAG pipeline includes de Bruijn genome assembly using SPAdes v.3.10.1 (121), and the bins were defined through distance clustering based on coverage and tetranucleotide signature using MetaBat v2 (122) and refined using GTDB-Tk (89). Genomes were annotated using PROKKA v1.13 (90), annotated through evidence from the nomenclature of the Consortium for Function Glycomics, eggNOG (v4.5) (123), KEGG (18 March 2013 release) (124), Pfam (v30.0) (125), and CAZy (2014 release) (126, 127). Similarity searches were performed for comparison with previously annotated enzymes or transporter proteins based on the accession numbers (36–38) using BLASTP and confirmed with COG, Pfam, and CAZy annotation evidence to ensure the integrity of the results. The 8 essential extracellular enzymes that are known to be required for the extracellular cleavage of HMOs before importing selected products of degradation were investigated (36–38), including 1,2-α-l-fucosidase (AfcA), 1,3/4-α-l-fucosidase (AfcB), 2,3/6-α-sialidase (SiaBb2), lacto-*N*-biosidase (LnbB and LnbX), the chaperone for LnbX (LnbY), β-1,4-galactosidase (BbgIII), and β-*N*-acetylglucosaminidase (BbhI). Five essential bacterial ABC transporters and homologs involved in the import of oligosaccharides were examined, which are known to show an exquisite specificity conferred by substrate binding protein (SBPs) for different HMO molecules (39), including the GNB/LNB (galacto-*N*-biose/lacto-*N*-biose I) transporter SBP (GltA), the FL transporter SBPs (FL1-BP and FL2-BP), and the LNnT transporter SBP (NahS). In addition to similarity searches on *Bifidobacterium* genomes and MAGs, we also confirmed the results by searching the metagenomic community gene content to verify that the target genes are not from species other than *Bifidobacterium*.

Metapangenomes were prepared using the MAGs constructed in this study and publicly available genomes under the species names B. breve (taxID 1685) and B. longum (taxID 216816) (https://doi.org/10.6084/m9.figshare.19709917). The metapangenome was constructed using anvi’o version 6.2 (128) according to the pangenome workflow (114). HGCs were identified in this set of genomes based on all-versus-all sequence similarity. Briefly, this workflow uses BLASTP to compute the average nucleotide identity (ANI) between all pairs of genes, uses the Markov cluster algorithm (MCL) (129) to generate homologous gene clusters, and aligns amino acid sequences using MUSCLE (130) for each gene cluster. Each gene was assigned as core or accessory according to the hierarchical clustering of the gene clusters. Sourmash version 3.3 (131) was used to compute ANIs across genomes. To count a gene as being present in the sample, it had to be of at least 50 reads mapping to at least one *Bifidobacterium* species genome, and the total abundance had to be at least 0.1% after normalizing over the total number of reads. For long-read data sequenced on the Pacific Biosciences Sequel II platform, quality control (QC) and assembly were performed using Canu-1.8 (86). The assemblies were assigned species names through BLAST to the RefSeq data set and confirmed with GTDB-Tk v1.1.0 (89). Genomes of the assemblies assigned to B. breve were aligned to reference B. breve genome JCM1192 using MAUVE aligner (132, 133).

### Code availability.

The R code for processing these sequences and the SAS code used in this statistical analysis have been deposited at https://github.com/igsbma/IP_microbiome/tree/main/statistical_analyses. Detailed information on sequences and annotation of the pangenome can be retrieved at https://github.com/igsbma/IP_microbiome/tree/main/pangenome.

### Data availability.

All metagenomic, metataxonomic, and genomic data were deposited in the NCBI database under BioProject accession number PRJNA774819 for open assessment. Illumina 16S rRNA V3-V4 gene amplicon and Pacific Biosciences full-length 16S rRNA gene data were deposited in the Sequence Read Archive under accession numbers SRX12805867 to SRX12806634. Data deposition includes samples of positive and negative controls in each plate. Metagenomic data using the Pacific Biosciences platform were deposited in the Sequence Read Archive under accession numbers SRR16598000 and SRR16598001. Metagenomic data using the Illumina platform were deposited in BioProject under accession numbers SRX12798907 to SRX12798933. The assembled genomes of B. breve were deposited in GenBank under accession numbers JAJGBR000000000 and JAJGBS000000000.
