# Supplementary material for: Highly Specialized Carbohydrate Metabolism Capability in Bifidobacterium Strains Associated with Intestinal Barrier Maturation in Early Preterm Infants
Source: mBio. 2022 Jun 13;13(3):e01299-22. doi: 10.1128/mbio.01299-22 (PMC9239261; doi:10.1128/mbio.01299-22)
Supplement: FIG S1 [file mbio.01299-22-s0001.pdf]

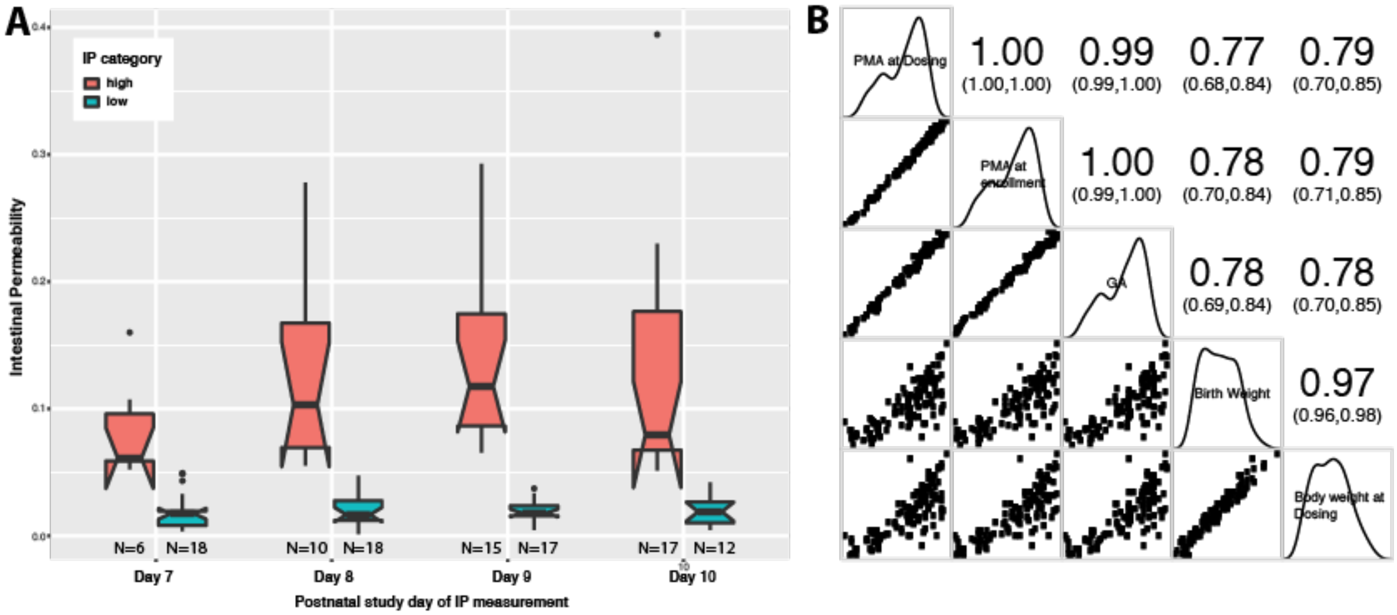

FIG S1 Intestinal permeability (IP) and cohort clinical information. **A**) Notched boxplot of IP for early preterm subjects (GA < 33 weeks gestation). Subjects were categorized by IP-measuring day between study day 7-10 and by IP category. The top and bottom of the box are the lower and upper quartiles, and the band near the middle of the box represents the median. The width of the notch can be used to roughly compare two distributions. For example, two distributions without overlapping notch regions can be roughly considered as being significantly different from each other (1). IP was measured by non-metabolized sugar probes lactulose and rhamnose. High IP was defined by a La/Rh ratio >0.05, as validated and applied previously (2). **B**) Correlation matrices visualization of the subjects' physiological age. R package Correlograms (corrgram) were used to visualize the correlation matrices. Pearson correlation method used to calculate correlation. **Abbr**: PMA at dosing: postmenstrual age calculated at the dosing day when IP was measured; PMA at enrollment: postmenstrual age at enrollment day taken place within 1-4 days post-birth; GA: gestational age; BW: birthweight; body weight at dosing: subject weight measured at the dosing day when IP was measured.

1. McGill R, Tukey JW, Larsen WA. 1978. Variations of Box Plots. *The American Statistician* 32:12–6
2. Saleem B, Okogbule-Wonodi AC, Fasano A, Magder LS, Ravel J, et al. 2017. Intestinal Barrier Maturation in Very Low Birthweight Infants: Relationship to Feeding and Antibiotic Exposure. *J Pediatr* 183:31-6 e1
