# Supplementary material for: Highly Specialized Carbohydrate Metabolism Capability in Bifidobacterium Strains Associated with Intestinal Barrier Maturation in Early Preterm Infants
Source: mBio. 2022 Jun 13;13(3):e01299-22. doi: 10.1128/mbio.01299-22 (PMC9239261; doi:10.1128/mbio.01299-22)
Supplement: TABLE S2 [file mbio.01299-22-s0008.docx]

| **TABLE S2. Dependence of demographic, obstetric, and neonatal characteristics with intestinal permeability (IP) using Hilbert-Schmidt Independence Criterion (HSIC)** | | | | |
| --- | --- | --- | --- | --- |
| **Factors** | **HSIC statistic** | **p-val** | **q-val** | **factor group** |
| **total amount of breastmilk feeding (mom's and donor's)** | 1.534 | 0.001 | 0.006 | Modifiable |
| **total amount of mom's own breastmilk feeding** | 1.461 | 0.001 | 0.006 | Modifiable |
| **gestational age** | 1.125 | 0.001 | 0.006 | Non-Modifiable |
| **postmenstrual age *** | 0.940 | 0.004 | 0.016 | Non-Modifiable |
| **body weight **** | 0.841 | 0.010 | 0.036 | Non-Modifiable |
| **birth weight** | 0.809 | 0.004 | 0.016 | Non-Modifiable |
| **duration of formula feeding (days)** | 0.748 | 0.024 | 0.070 | Modifiable |
| **total amount of formula feeding** | 0.694 | 0.032 | 0.085 | Modifiable |
| **duration of antibiotics uses (days)** | 0.676 | 0.018 | 0.058 | Modifiable |
| **duration of mom's own breastmilk feeding (days)** | 0.579 | 0.039 | 0.096 | Modifiable |
| **duration of ampicillin use (days)** | 0.570 | 0.052 | 0.119 | Modifiable |
|  | | | | |
|  | ***Postmenstrual age calculated as the sum of gestational age and postnatal days at the time when IP was measured.** | | | |
|  | **** body weight was the weight measured on the day when IP was measured.** | | | |
