# Supplementary material for: Highly Specialized Carbohydrate Metabolism Capability in Bifidobacterium Strains Associated with Intestinal Barrier Maturation in Early Preterm Infants
Source: mBio. 2022 Jun 13;13(3):e01299-22. doi: 10.1128/mbio.01299-22 (PMC9239261; doi:10.1128/mbio.01299-22)
Supplement: FIG S2 [file mbio.01299-22-s0002.pdf]

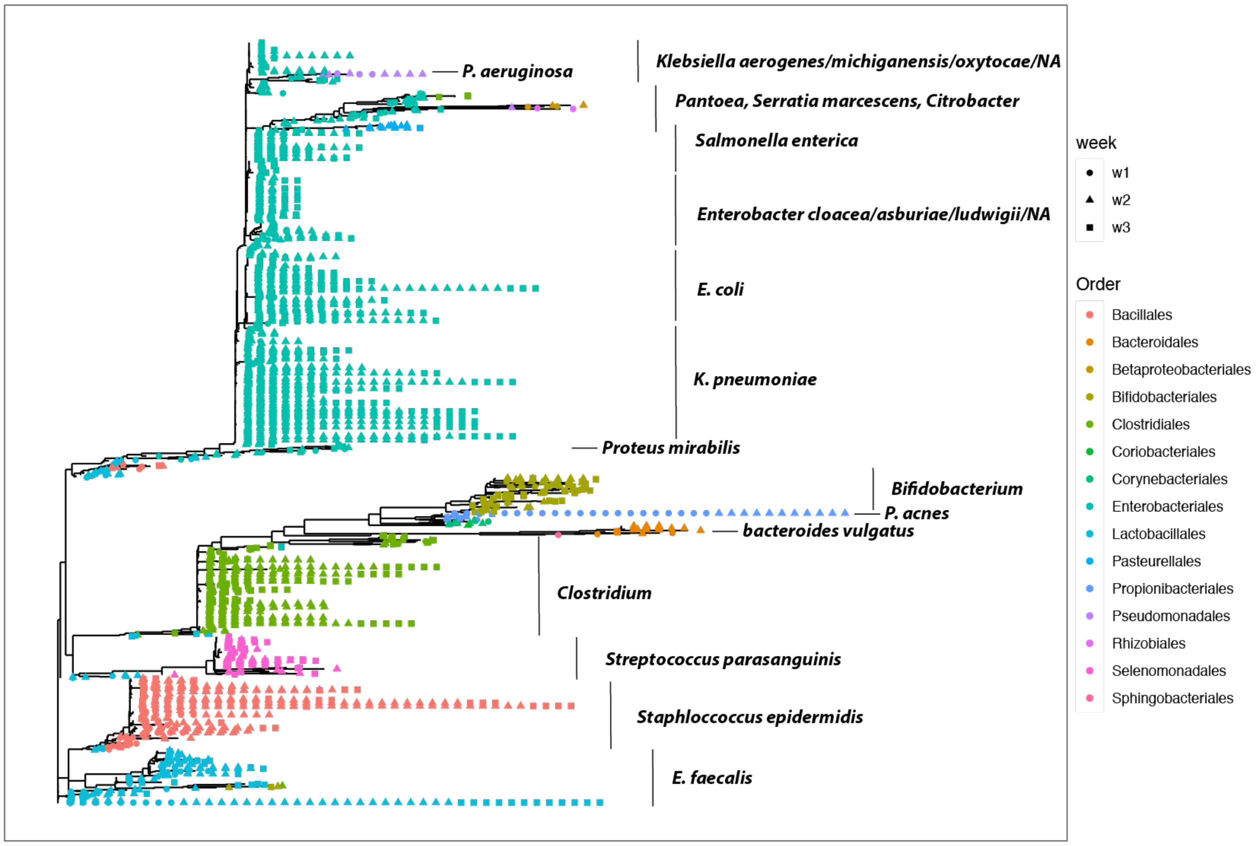


**FIG S2** Phylogenetic tree of all ASVs of full-length 16S rRNA gene sequenced on Pacific Biosciences Sequel II platform. Package raxml (v8.0.0) (1) was used to construct the phylogeny, Phyloseq R package (2) was used to display the phylogeny.


1. Stamatakis A. 2014. RAxML version 8: a tool for phylogenetic analysis and post-analysis of large phylogenies. *Bioinformatics* 30:1312-3
2. McMurdie PJ, Holmes S. 2013. phyloseq: an R package for reproducible interactive analysis and graphics of microbiome census data. *PLoS One* 8:e61217
