# Supplementary material for: Highly Specialized Carbohydrate Metabolism Capability in Bifidobacterium Strains Associated with Intestinal Barrier Maturation in Early Preterm Infants
Source: mBio. 2022 Jun 13;13(3):e01299-22. doi: 10.1128/mbio.01299-22 (PMC9239261; doi:10.1128/mbio.01299-22)
Supplement: FIG S3 [file mbio.01299-22-s0003.pdf]

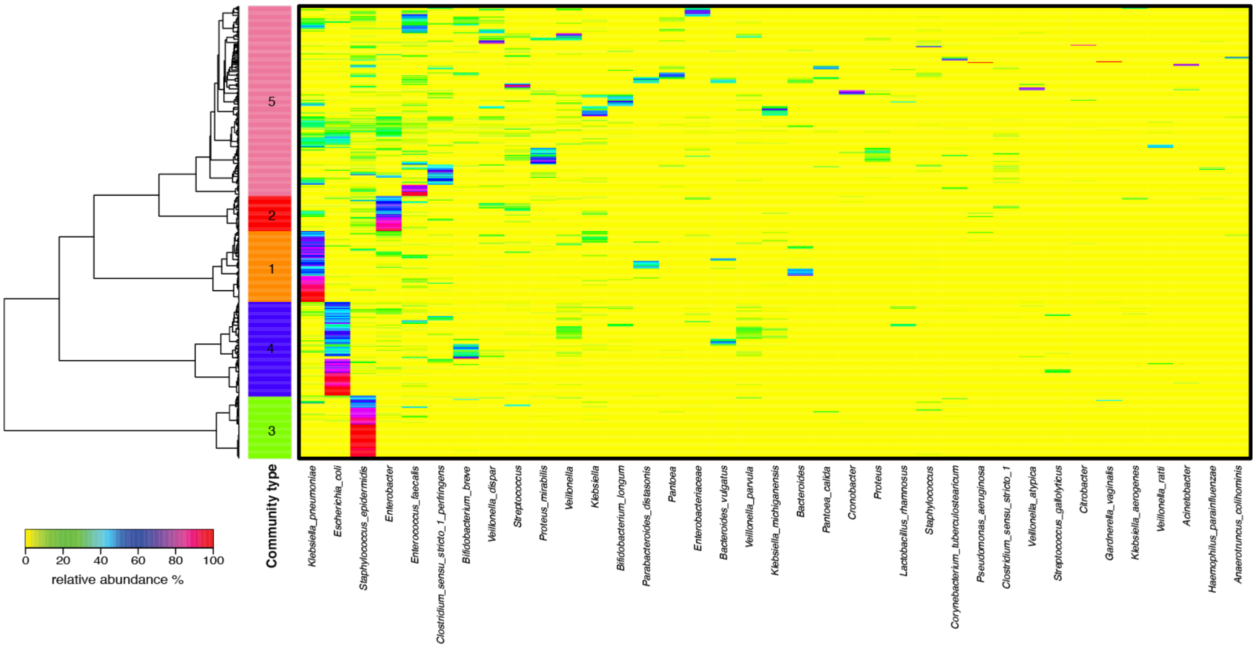

FIG S3 Heatmap of the 50 most abundant intestinal bacterial taxa relative abundance in samples collected from 113 preterm infants enrolled in the study. The fecal microbiota was characterized by high-throughput sequencing of the V3-V4 variable regions of 16S rRNA genes. Ward linkage clustering was used to cluster samples based on their Jensen-Shannon distance calculated in vegan package in R (1). The number of clusters was validated using gap statistics implemented in the *cluster* package in R (2) by calculating the goodness of clustering measure.

1. Oksanen J, Blanchet FG, Kindt R, Legendre P, Minchin PR, et al. 2011. vegan: Community Ecology Package. *R package version 2.0-2.*
2. Maechler M. 2016. *cluster: "Finding Groups in Data": Cluster Analysis Extended Rousseeuw et al.*
