# Supplementary material for: Highly Specialized Carbohydrate Metabolism Capability in Bifidobacterium Strains Associated with Intestinal Barrier Maturation in Early Preterm Infants
Source: mBio. 2022 Jun 13;13(3):e01299-22. doi: 10.1128/mbio.01299-22 (PMC9239261; doi:10.1128/mbio.01299-22)
Supplement: FIG S4 [file mbio.01299-22-s0004.pdf]

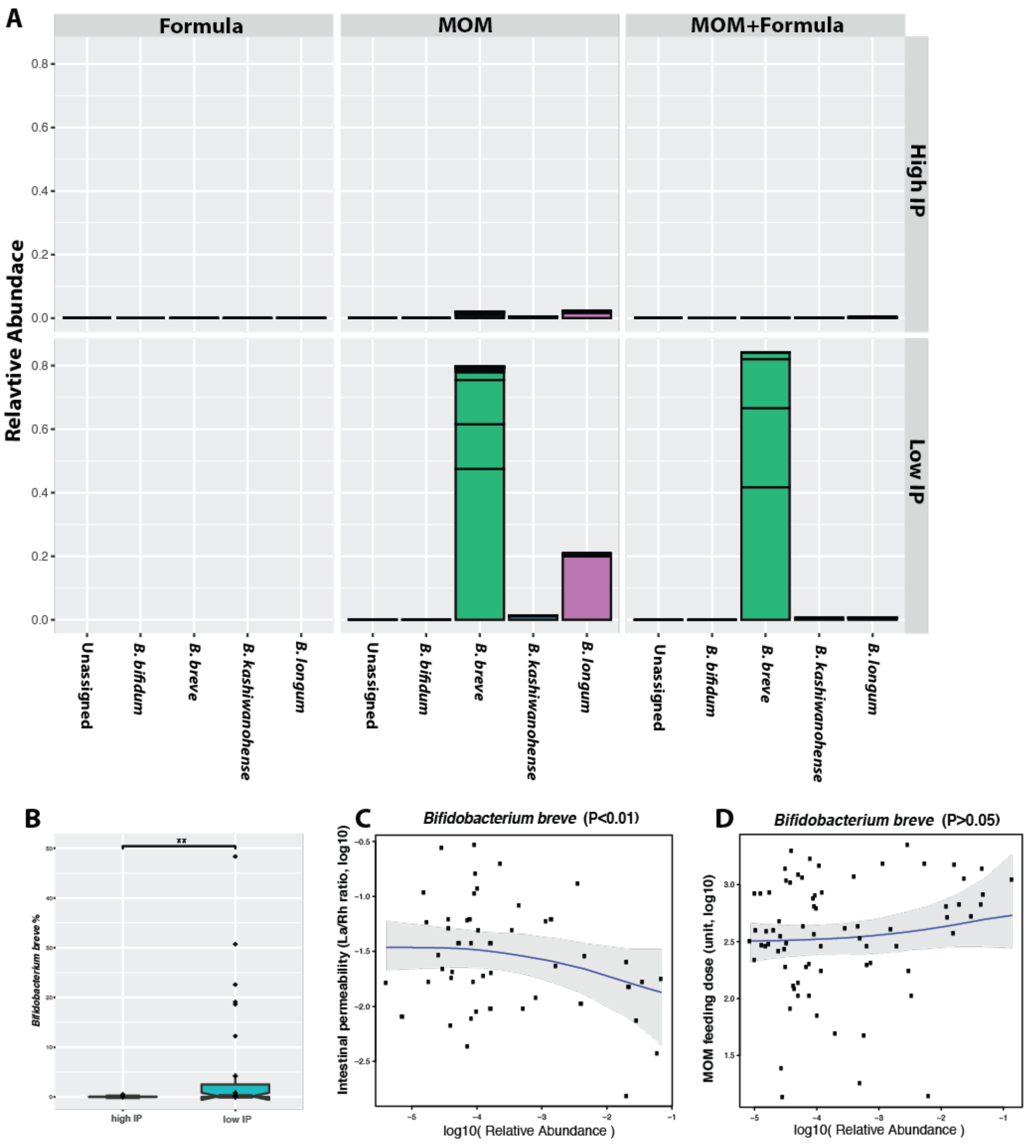

FIG S4 Information on bifidobacterial abundance and intestinal permeability (IP). **A**) Relative abundance of bifidobacterial bacterial groups stratified by feeding types. Phyloseq R package (v1.38.0) (1) was used to generate the barplot. **B**) The relative abundance of *B. breve* between high-IP and low-IP groups. Dependence between **C**) IP or **D**) MOM feeding dose and the log relative abundance of *B. breve*. An adaptive spline logistic regression model implemented in spmrf R package (2) was applied to the phylotypes present in at least 15% of all samples. Bayesian goodness-of-fit p-value implemented in R package rstan (3) was used to access the significance of the association between phylotypes and investigated factors.

1. McMurdie PJ, Holmes S. 2013. phyloseq: an R package for reproducible interactive analysis and graphics of microbiome census data. *PLoS One* 8:e61217
2. Faulkner JR, Minin, V. 2018. Locally adaptive smoothing with Markov random fields and shrinkage priors. *Bayesian Analysis* 13:225-52
3. Team SD. 2018. RStan: the R interface to Stan. R package version 2.17.3.
