# Supplementary material for: Highly Specialized Carbohydrate Metabolism Capability in Bifidobacterium Strains Associated with Intestinal Barrier Maturation in Early Preterm Infants
Source: mBio. 2022 Jun 13;13(3):e01299-22. doi: 10.1128/mbio.01299-22 (PMC9239261; doi:10.1128/mbio.01299-22)
Supplement: FIG S5 [file mbio.01299-22-s0005.pdf]

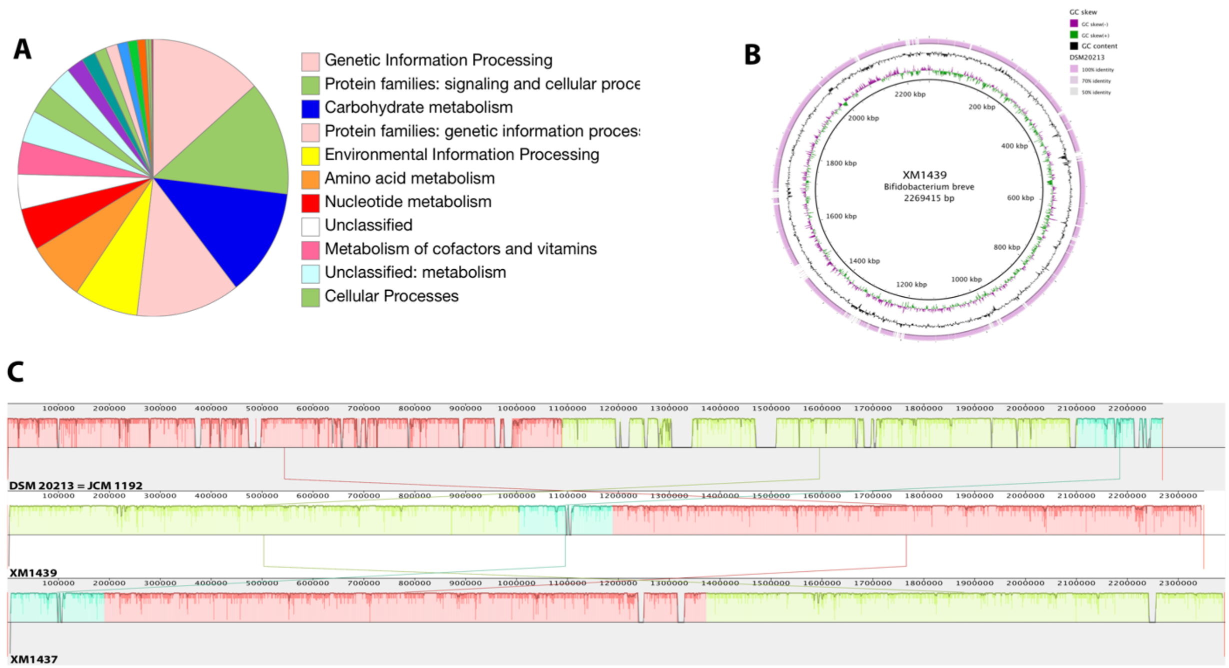

FIG S5 Metapangenome of *Bifidobacterium breve*. The 26 *in-house B. breve* MAGs was supplemented with 107 published genomes (https://doi.org/10.6084/m9.figshare.19709917.v2 **A**) and our 4 *B. longum* MAGs was supplemented with 310 published genomes (https://doi.org/10.6084/m9.figshare.19709917.v2 **B**) for pangenome construction following pangenome workflow (1). *B. breve* pangenome was displayed using anvi'o *vers* 6.2 (2). BLASTP was used to compute ANI identity between all pairs of genes. Markov Cluster Algorithm (MCL) (3) was used to generate homologous gene clusters (HGCs). Amino acid sequences of each HGC were aligned using MUSCLE (4). HCG was assigned to core, accessory or dispensable according the hierarchical clustering of the gene clusters. Detail of each HGC was in https://doi.org/10.6084/m9.figshare.19709917.v2 **C**. Sourmash vers 3.3 (5) was used to compute Average nucleotide identity (ANI) across genomes. The source indicates the isolated origin of the genome, and genomes of the same subject are indicated in the same cohort.

1. Muret EA. 2020. An anvi'o workflow for microbial pangenomics. http://merenlab.org/2016/11/08/pangenomics-v2/.
2. Delmont TO, Eren AM. 2018. Linking pangenomes and metagenomes: the Prochlorococcus metapangenome. PeerJ 6:e4320.
3. Enright AJ, Van Dongen S, Ouzounis CA. 2002. An efficient algorithm for large-scale detection of protein families. Nucleic Acids Res 30:1575-84.
4. Edgar RC. 2004. MUSCLE: multiple sequence alignment with high accuracy and high throughput. Nucleic Acids Res 32:1792-7.
5. Pierce NT, Irber L, Reiter T, Brooks P, Brown CT. 2019. Large-scale sequence comparisons with sourmash. F1000Res 8:1006.
