# Supplementary material for: Highly Specialized Carbohydrate Metabolism Capability in Bifidobacterium Strains Associated with Intestinal Barrier Maturation in Early Preterm Infants
Source: mBio. 2022 Jun 13;13(3):e01299-22. doi: 10.1128/mbio.01299-22 (PMC9239261; doi:10.1128/mbio.01299-22)
Supplement: FIG S6 [file mbio.01299-22-s0006.pdf]

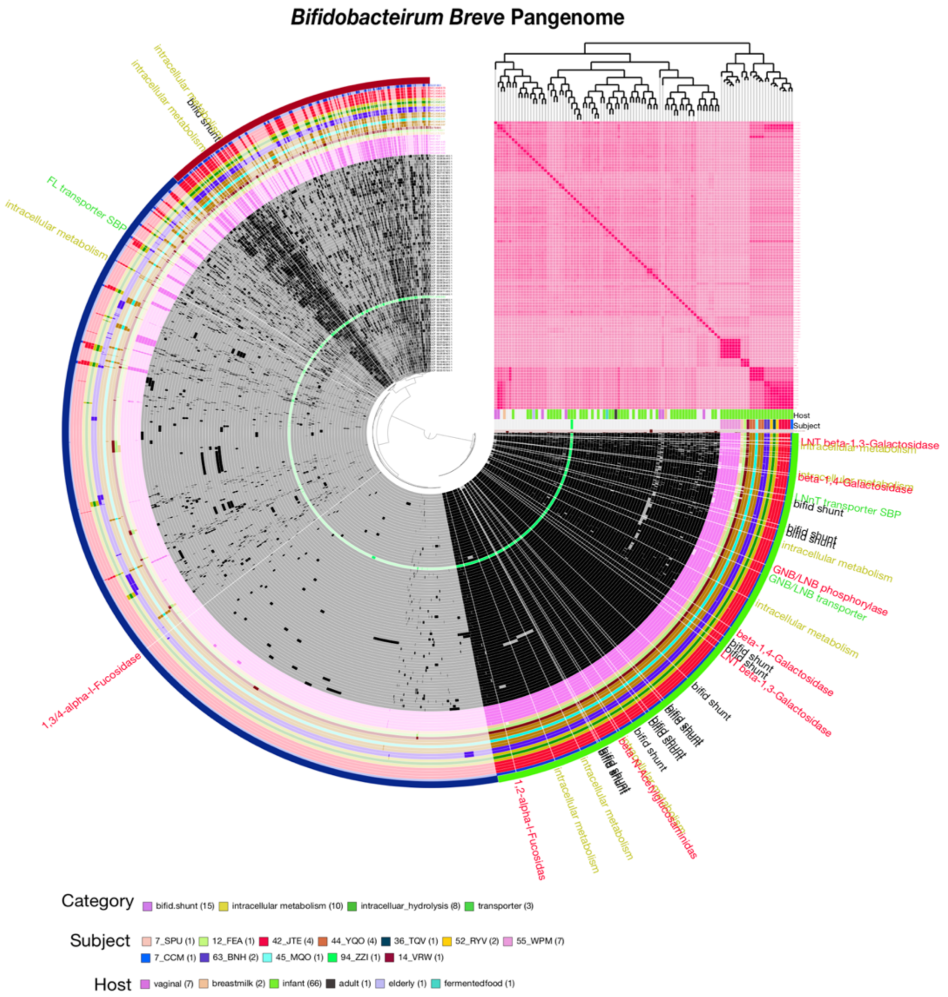

FIG S6 The complete *B. breve* genome reconstructed in this study. Metagenomic sequencing of the two selected fecal samples was performed using the Pacific Bioscience Sequel II platform, followed by assembly using Canu v1.8 (1) and deconvolution using BLASTN of the assembly. This complete genome was 2.34M in size (https://doi.org/10.6084/m9.figshare.19709923.v1**, Table S5C**), similar to median *B. breve* genome size of 2.33M reported on NCBI. **A**) KEGG 2013-03-18 release (2) to characterize functional categories of *B. breve* XM1439. **B**) Circular genome display of *B. breve* XM1439, generated by BLAST Ring Image Generator (BRIG) (2011 June vers) (3). **C**) Genome alignment of *B. breve* genome 1439, 1437 using MAUVE (4) using *B. breve* DSM20213 as the reference genome.

1. Koren S, Walenz BP, Berlin K, Miller JR, Bergman NH, Phillippy AM. 2017. Canu: scalable and accurate long-read assembly via adaptive k-mer weighting and repeat separation. Genome Res 27:722-736.
2. Kanehisa M, Goto S, Sato Y, Furumichi M, Tanabe M. 2012. KEGG for integration and interpretation of large-scale molecular data sets. Nucleic acids research 40:D109-14.
3. Alikhan NF, Petty NK, Ben Zakour NL, Beatson SA. 2011. BLAST Ring Image Generator (BRIG): simple prokaryote genome comparisons. BMC Genomics 12:402.
4. Rissman AI, Mau B, Biehl BS, Darling AE, Glasner JD, Perna NT. 2009. Reordering contigs of draft genomes using the Mauve aligner. Bioinformatics 25:2071-3.
